# Relational continuity may give better clinical outcomes in patients with serious mental illness – a systematic review

**DOI:** 10.1186/s12888-023-05440-1

**Published:** 2023-12-18

**Authors:** Ingemar Engström, Lars Hansson, Lilas Ali, Jenny Berg, Mirjam Ekstedt, Sven Engström, Maja Kärrman Fredriksson, Jan Liliemark, Per Lytsy

**Affiliations:** 1 University Health Care Center, Faculty of Medicine and Health, Örebro University, Örebro, SE-701 82 Sweden; 2 Department of Health Sciences, Lund University, Lund, Sweden; 3 Institute of Health and Care Sciences, University of Gothenburg, Gothenburg, Sweden; 4 SBU – Swedish Agency for Health Technology Assessment and Assessment of Social Services, Stockholm, Sweden; 5 Department of Health and Caring Sciences, Linnaeus University, Kalmar, Sweden; 6 Futurum, Region Jönköping County, Jönköping, Sweden

**Keywords:** Relational continuity, Continuity of care, Serious mental Illness, Mortality, Health care utilization

## Abstract

**Background:**

Continuity of care is considered important for results of treatment of serious mental illness (SMI). Yet, evidence of associations between relational continuity and different medical and social outcomes is sparse. Research approaches differ considerably regarding how to best assess continuity as well as which outcome to study. It has hitherto been difficult to evaluate the importance of relational continuity of care. The aim of this systematic review was to investigate treatment outcomes, including effects on resource use and costs associated with receiving higher relational continuity of care for patients with SMI.

**Methods:**

Eleven databases were searched between January 2000 and February 2021 for studies investigating associations between some measure of relational continuity and health outcomes and costs. All eligible studies were assessed for study relevance and risk of bias by at least two independent reviewers. Only studies with acceptable risk of bias were included. Due to study heterogeneity the synthesis was made narratively, without meta-analysis. The certainty of the summarized result was assessed using GRADE. Study registration number in PROSPERO: CRD42020196518.

**Results:**

We identified 8 916 unique references and included 17 studies comprising around 300 000 patients in the review. The results were described with regard to seven outcomes. The results indicated that higher relational continuity of care for patients with serious mental illness may prevent premature deaths and suicide, may lower the number of emergency department (ED) visits and may contribute to a better quality of life compared to patients receiving lower levels of relational continuity of care. The certainty of the evidence was assessed as low or very low for all outcomes. The certainty of results for the outcomes hospitalization, costs, symptoms and functioning, and adherence to drug treatment was very low with the result that no reliable conclusions could be drawn in these areas.

**Conclusions:**

The results of this systematic review indicate that having higher relational continuity of care may have beneficial effects for patients with severe mental illness, and no results have indicated the opposite relationship. There is a need for better studies using clear and distinctive measures of exposure for relational continuity of care.

**Supplementary Information:**

The online version contains supplementary material available at 10.1186/s12888-023-05440-1.

## Summations

- Relational continuity of care (CoC) is much called for in mental health care for patients with serious mental illness (SMI).
- This is a systematic review investigating the associations between measures of CoC and different clinical and social outcomes.
- We found some evidence that having higher relational CoC may be advantageous for care of patients with SMI, especially in association with premature deaths, suicide, emergency department visits and quality of life.

## Limitations

- The results are mainly based on studies using observational study designs.
- Meta-analysis was not possible due to large methodological heterogeneity.
- Residual confounding affects the certainty of the evidence and cannot be ruled out completely.

## Background

Serious mental illness (SMI) is a term that usually refers to persons diagnosed with schizophrenia, bipolar disorder or major depression [1]. Patients with SMI have a reduced quality of life [2], high rates of comorbidity [3], shortened life expectancy [4], and high rates of unplanned care needs [5]. SMI often goes with substantial functional impairment in domains like employment, housing and social integration.

The wide scope of psychiatric symptoms and social difficulties connected with SMI means that it is important for persons with SMI to have good access to health providers who can give comprehensive treatment and support over time. Interventions for patients with SMI must be based on good relationships between them and staff [6]. Continuity of care (CoC) has for many years been stated as an essential component of health care for patients with SMI [7].

There has been a steady increase in studies about the benefits of CoC. A number of qualitative studies have shown that CoC is highly appreciated by patients [8]. Quantitative studies focused on the association between CoC and clinical outcomes have shown more diverse results [9]. To date, evidence has been mixed on whether relational continuity improves outcomes for people with SMI.

There may be several different reasons for the diversity of research results in this field. One reason may be that the concept CoC is multidimensional and used with different implications, both regarding the process of care and from the perspective of the patients [10]. This has been suggested in theoretically based studies as well as in factor analysis derived studies [11].

Despite the importance placed on providing continuity, its definitions differ [12]. Another reason may be that the methods used for studying the question vary considerably between studies. A third reason may be a variation concerning what parts of the health system that are included in the studies. An example of this is that some studies only study effects on mental health care utilization whereas other studies also cover somatic health care including emergency department visits.

There seems to be consensus that CoC is a construct that is broadly defined as the long-term delivery of care over time which is coordinated among services to appropriately meet patient’s current needs [13]. Continuity is perceived by both the patients and staff as care that is comprehensive, consistent and connected [12]. There is also consensus that CoC is a multi-dimensional construct [14], especially within mental health services that usually involves more complex, integrated and coordinated care pathways [12].

A distinction between three different types of continuity has been a commonly used point of departure for several researchers in this field [14]. The first type called *informational continuity* focuses on systems for longitudinal information about past events and personal circumstances that may be important for current care givers. The second type called *management continuity* (sometimes called coordination continuity or treatment continuity) focuses on the ability of the health care system as a whole to provide and coordinate treatment modalities responsive to the changing needs of the patient. The third type called *relational continuity* (sometimes called interpersonal continuity) focuses on the longitudinal relationship between a patient and one or more health care providers that extends beyond specific episodes of illness [15]. The relationship may be with a few persons or a multidisciplinary team, which is often the case in mental health care. In certain treatment modalities, such as different forms of psychotherapy, continuity with the therapist is of special importance [6]. Relational continuity is often considered a cornerstone for organizing modern health care, which may counteract the risks of fragmentation that may be a consequence of high specialization [13]. Both patients and professionals tend to endorse the importance of CoC, which nevertheless not often is reached for various reasons. It is well known that discontinuity of care is a major source of patient dissatisfaction and disengagement [16, 17].

Since CoC is a multidimensional construct with different focus, a considerable number of instruments have been used in medical research [12]. There are several instruments specifically developed for measuring relational continuity. The following instruments have been used in the studies that were included in this systematic review: Alberta Continuity of Services Scale-Mental Health (ACSS-MH) [18], Continuity of Care Index (COC Index) [19], Continuity of Care – User Measure (CONTINU-UM) [20], Modified Modified Continuity Index (MMCI) [21], Sequential Nature of Provider Continuity (SECON) [22], Usual Provider of Care (UPC) [23]. Some of these instruments were developed within primary or somatic care, whereas others were specifically developed to assess continuity in mental care services [12]. Some of the studies used specific measures, for example the number of staff that a patient has met or the number of unique prescribers of drugs over a specific time period. Common variables have included aspects of relationship duration, density of visits, dispersion of providers, or sequence of providers [23]. Detailed information on the methods used in the reviewed studies is provided in Additional file 5.

The aim of this systematic review was to investigate treatment outcomes, including effects on resource use and costs, associated with receiving higher relational continuity of care for patients with SMI.

## Methods

The review was conducted at the Swedish Agency for Health Technology Assessment and Assessment of Social Services, SBU, following a protocol pre-registered on the International prospective register of systematic reviews (PROSPERO: CRD42020196518). Results of the other study population (asthma/COPD) mentioned in the protocol have been reported separately [24]. It was not possible to perform a meta-analysis due to considerable heterogeneity in the included studies. The goal was, thus, to perform a synthesis without meta-analysis to summarize clinical outcomes associated with higher relational continuity of health care for patients with SMI. The certainty of the evidence was assessed using the Grading of Recommendations Assessment, Development, and Evaluation (GRADE) system [25], aiming at being supportive when going from evidence to recommendations [26].

### Research question and selection criteria

The research question and the inclusion criteria were formulated using the PICO/PECO structure. Necessary attributes of the target population were a diagnosis of SMI (defined as schizophrenia, bipolar disorder or major depression) and to be at least 18 years of age. In the case of mixed populations, results were included if relevant subpopulations were reported or if the majority of the participants had any of these diagnoses.

The exposure had to be clearly defined and relevant to relational continuity of care and had to use an established continuity index or measure of duration, density, dispersion, sequencing, fragmentation, or discontinuation of regular care to either a specified person or a team of health care professionals. The exposure should have been present for at least 12 months. Intervention studies were required to alter a dimension of continuity of care. Each included study was checked in relation to these inclusion criteria using consensus in the project group. Both experimental controlled studies and observational studies (cohort and register studies) were considered for inclusion.

The main outcomes were mortality, morbidity (symptoms and functioning), health care utilization (emergency department visits, hospitalizations) and health care costs. Additional outcomes were adherence to prescribed medical treatment, relevant laboratory measures and subjective measures such as patient satisfaction and quality of life, if measured by validated instruments. Studies using qualitative methodology were not included in the review.

Included studies had to be published in an international peer-reviewed journal in English from year 2000 and forward. The time restriction was chosen due to the fact that results from older studies may be uncertain since mental health care organizations and the content of care changes over time.

### Literature search

An information specialist developed, tested and further developed a search strategy with the assistance of the researchers in the project group. Blocks of search terms about the populations and the exposure ‘continuity of patient care’ were used in subject headings and in titles and abstracts. Literature searches were performed in the following databases: CINAHL (Cumulative Index to Nursing and Allied Health Literature), Cochrane Library, Clinicaltrials.gov, AHRQ (Agency for Healthcare Research and Quality), CRD (Centre for Reviews and Dissemination Database, Embase (Excerpta Medica dataBASE), Epistemonikos, KSR Evidence (Kleijnen Systematic Reviews), Medline, NICE Evidence Search (The National Institute for Health and Care Excellence), Prospero, APA PsycINFO and Scopus. The search was performed in May to June 2020 and was updated in early February 2021. Grey literature, books and conference abstracts were not considered. The full search strategy is provided in Additional file 1.

### Screening and assessment of relevance

All titles and abstracts were screened in relation to the inclusion criteria independently done by two researchers using the Covidence platform (covidence.org). Disagreements were resolved through discussion in the full research group, and if questions remained, studies were included to be read in full length. The selected articles were then read independently by two researchers with expert knowledge in the field of mental health to determine their relevance in relation to the set inclusion criteria. Disagreements were discussed in the larger research group. When the group was uncertain, the article was included to not lose too much information. The implications of any indirectness were handled later when rating the certainty of evidence.

### Assessment of risk of bias

An instrument was developed to assess risk of bias in observational studies. It was based on the preliminary tool for assessing risk of bias of exposure studies, ROBINS-E, and other risk of bias assessment tools used at the SBU. The reason not to use the full ROBINS-E tool was that there, at the time, were concerns about its application [27]. The instrument covered different domains that may affect risk of bias: confounding, exposure, dropout, measurement and analysis of outcomes, reporting, and conflicts of interests. Overall risk of bias for each study was classified as low, moderate, high or unacceptably high. A translated version of the instrument used to assess risk of bias in observational studies is available in Additional file 2.

### Data analysis, synthesis, and rating of the certainty of evidence

Results were extracted by one of two authors (PL, JB) and was quality assured by one of three mental health experts in the group (IE, LH, LA). Disagreements were resolved by discussions in the project group. Data extracted included study design, country, population, setting, participants’ age and sex, measurement of exposure or intervention, measurement of outcome, type of statistical analysis, confounders/covariates in analysis and main results.

Exposure for continuity was measured and analysed in many ways, which made meta-analyses impossible. Instead, a summarizing result regarding main and additional outcomes was synthesized narratively. The certainty of evidence was rated as high, moderate, low or very low, according to the GRADE framework in which five domains were considered: risk of bias, inconsistency, indirectness, imprecision and publication bias [28].

In the absence of meta-analytic confidence intervals, precision were assessed using the number of participants and events for overall results as well as reflecting on precision in included studies reporting their results with confidence intervals. Studies with high risk of bias were included to not lose information in an area with potentially few studies per outcome, and any indirectness of the continuity measure was taken into consideration when rating the certainty of the evidence. Because of a potential risk of residual confounding, all studies using observational data for causal analysis were considered to have at least moderate risk of bias.

### Material

**Fig. 1:**
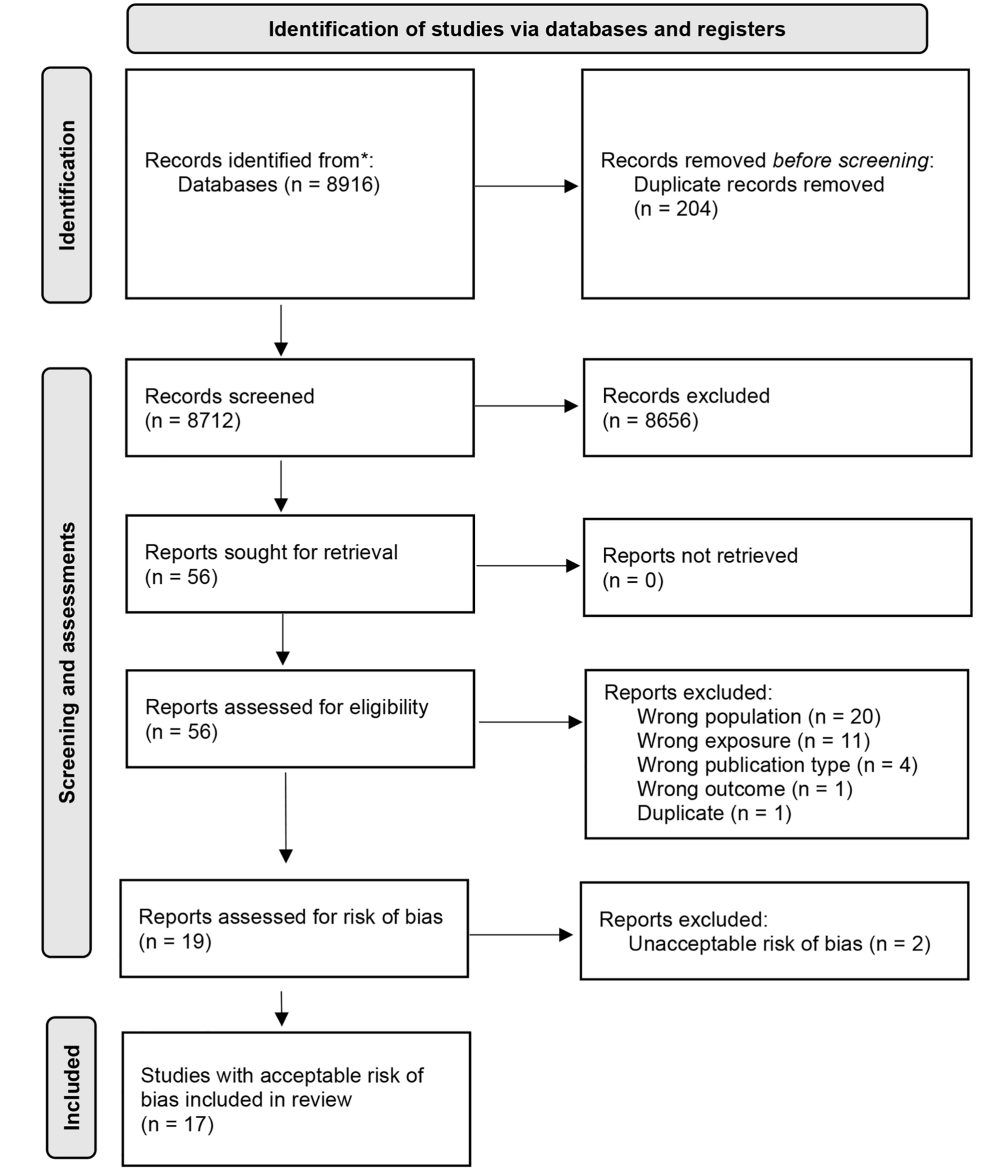
PRISMA flow chart

We identified 8 712 unique references that were screened for inclusion of which 56 articles were read in full text. Thirty-seven of these were excluded for different reasons. Nineteen articles fulfilled the inclusion criteria, of which two were later excluded because of an unacceptable risk of bias. Of the 17 included articles [9, 29–44], 16 were based on observational data, mainly retrospective cohort studies, and one was a prospective natural experimental study. Two articles were based on the same study population but reported different outcomes [29, 42]. Of the 17 included articles, eleven were assessed to have moderate risk of bias and six to have high risk of bias. The identification, selection and outcome of risk of bias assessments of included studies is shown in Fig. 1 (flow chart). The risk of bias assessments for included studies and reasons for exclusion of the other studies are shown in Additional files 3 and 4, respectively. A summary of characteristics of the included studies and ratings of evidence are presented in Table 1. Detailed information about the studies included is provided in Additional file 5.

**Table 1:** Summary of study characteristics of the included studies. Results and further details are available in Additional file 5

Author Year	Study type Country	Population n	Measure of exposure (continuity)	Outcome(s)	Overall risk of bias
**Adair et al.** **2005**	Prospective cohort study Canada, Alberta	SMI n = 486 65% mood disorders 35% psychotic disorders	Alberta Continuity of Services Scale (ACSS-MH)	EQ-5D	High
**Adnanes et al.** **2019**	National cross-sectional survey Norway	SMI or non-SMI patients receiving specialist outpatient psychiatric treatment n = 155 SMI n = 835 non-SMI	Perception of COC using the CONTINUUM measure	QOL measured with the Manchester Short Assessment of Quality of Life (MANSA) questionnaire	Moderate
**Bindman et al.** **2000**	Prospective cohort study England, south London	SMI n = 100 91% psychotic illness	Contact with number of community “keyworkers” over a period of time	BPRS (Brief Psychiatric Rating Scale) HoNOS (Health of the Nation Outcome Score) GAF (Global Assessment of Functioning)	Moderate
**Catty et al.** **2013**	Prospective cohort study England	Psychotic disorders n = 180	User rated overall “experienced” continuity using the CONTINUUM measure	BPRS (Brief Psychiatric Rating Scale GAF (Global Assessment of Functioning) Quality of Life - MANSA and SEQoL	High
**Chien et al.** **2000**	Register study USA, Maryland	Schizophrenia n = 351	Continuity of care (COC) Usual provider continuity (UPC) Sequential continuity (SECON)	Medicaid costs Lehman Quality of Health	Moderate
**Conti et al. 2012**	Register study Italy, Lombardy	SMI n = 11 797 42% schizophrenia 8% bipolar disorder 50% depression	Continuity of Care defined as receiving at least one psychiatric contact every 90 days	Lack of persistence with pharmacological treatment defined as a gap of at least 30 days between subsequent medication fills	Moderate
**Desai et al. 2005**	Register study from the VA health system USA	SMI with or without alcoholism, substance abuse or PTSD n = 121 933	Continuity of outpatient of care measured as the number of two-month periods in the six months after discharge in which the patient had at least two outpatient visits for his or her primary discharge diagnosis (range = 0–3)	Suicide	Moderate
**Farley et al. 2011**	Register study USA, North Carolina	Schizophrenia n = 7 868	Number of unique prescribers of medication for schizophrenia	Adherence to medication measured by the medication possession ratio (MPR) from Medicaid data	Moderate
**Giacco et al. 2018**	Prospective natural experiment Follow-up 1 year Belgium, England, Germany, Italy, Poland	Psychiatric in-patients 41% psychotic disorders 49% mood disorders 18% other disorders n = 7 302	Personal continuity, i.e., a patient is under the care of the same psychiatrist for in- and out- patient treatment; or specialization, i.e., a patient is under the care of different psychiatrists for in- and out-patient treatment.	Readmission to hospital within one year following the index admission	Moderate
**Hoertel et al. 2014**	Observational study France	Patients who visited a psychiatrist at least twice over six months n = 114 515 diagnosis available for n = 2 863 17% schizophrenia 29% major depressive disorder 10% bipolar disorder 38% personality disorder	COC Index	All-cause mortality	Moderate
**Kaltsidis et al.** **2020**	Retrospective observational study Canada, Quebec	Patients visiting emergency department for mental health reasons n = 210	Exploring predictors for ED visits based on predisposing, enabling and needs factors One of the enabling factors was a regular source of care over the 12 months prior to the research interview at the emergency department	Number of visits to the emergency department for mental health reasons over the 12 months prior to the index visit	Moderate
**Macdonald et al.** **2019**	Register study England, south London	Schizophrenia or delusional disorders 83% schizophrenia 17% delusional disorders n = 5 552	Modified Continuity Index (MMCI) measuring the number of teams caring for the patient over time	Health of the Nation Outcomes Scales (HoNOS)	Moderate
**Mitton et al.** **2005**	Observational study Alberta, Canada	SMI n = 486 65% mood disorders 35% psychotic disorders	Alberta Continuity of Services Scale (ACSS-MH)	Quality of Life (EQ-5D) Community functioning (Multnomah Community Ability Scale) Administrative data concerning service use events and health care costs	Moderate
**Puntis et al.** **2016**	Prospective cohort study England	Patients with psychosis diagnosis who were to be discharged from compulsory hospital treatment n = 323	Average gap between face-to-face contacts Number of 60-day gaps without contact Number of different mental health professions seen Number of care coordinators Number of psychiatrists	Readmission to hospital Time to readmission Number of days in hospital	Moderate
**Ride et al.** **2019**	Observational study England	SMI 53% schizophrenia and other psychoses 35% bipolar disorder 12% both diagnoses n = 19 324	Family physician relational continuity measured by: COC Continuity of Care UPC Usual Provider Care SECON Sequential Continuity	Emergency presentations Unplanned admissions for SMI Ambulatory care-sensitive conditions (ACSC)	Moderate
**van der Lee et al. 2016**	Retrospective register-based cohort study The Netherlands	Schizophrenia n = 7 392	The number of follow-up years of elective psychiatric care	Acute treatment events Inpatient care and somatic care Medical costs	High
**Watkins et al.** **2016**	Retrospective cohort study USA	Co-occurring mental illness (schizophrenia, bipolar disorder type 1, PTSD or depression) and substance use disorder n = 144 045	Continuous care over time defined as receiving ≥ 1 visit each quarter over a one-year period from any type of provider	Mortality after 12 and 24 months after the end of the observation period (main outcome) Avoidable excessive mortality number	Moderate

Notes: COC = Continuity of Care; ED = emergency department; EQ-5D = Euro Quality of Life 5 dimensions

Ten of the studies were performed in Europe (five from England, one each from France, Italy, the Netherlands and Norway, and one using data from four European countries) and seven studies in North America (three from Canada and four from the US). In total, the studies cover around 300 000 patients with SMI. Some of the studies were large register-based studies, whereas others were fairly small clinical studies.

## Results

The studies were quite different with respect to exposure as well as outcomes and have used different ways of analysing the material statistically. The outcomes were categorized into summarized outcomes as follows: mortality/suicide (two studies), hospitalizations (four studies), emergency department visits (three studies), costs (two studies), symptoms and functioning (three studies), adherence to pharmacotherapy (two studies) and quality of life (four studies). Summarized results and evidence ratings are presented in Table 2.

**Table 2:** Summarized results and evidence ratings

Outcome	Number of studies/ participants (n)	Summarized result	Certainty of evidence according to GRADE	Reasons for reduced certainty of the evidence
**Mortality**	3 n = 267 667	Higher relational continuity of care for patients with SMI may prevent premature mortality/suicide	Low	Risk of bias – 1 Indirectness – 0.5 Inconsistency – 0.5
**Hospitalization**	4 n = 34 341	Higher relational continuity of care for patients with SMI may lower the risk of hospitalization	Very low	Risk of bias – 1 Indirectness – 1 Imprecision – 1
**Emergency department visits**	3 n = 26 926	Higher relational continuity of care for patients with SMI may reduce the risk of emergency department visits	Low	Risk of bias – 1 Indirectness – 1
**Costs**	3 n = 8 229	Higher relational continuity of care for patients with SMI may lower health care costs	Very low	Risk of bias – 1 Indirectness – 1 Inconsistency – 1
**Symptoms and functions**	3 n = 5 832	It is not possible to say whether higher relational continuity of care can improve symptoms and functions in patients with SMI	Very low	Risk of bias – 2 Indirectness – 1 Imprecision – 1
**Adherence to pharmaco-logical treatment**	2 n = 19 765	It is not possible to say whether relational continuity of care affects adherence to pharmacological treatment for patients with SMI	Very low	Risk of bias – 1 Indirectness – 1 Inconsistency – 1
**Quality of life**	4 n = 2 007	Higher relational continuity of care for patients with SMI may affect quality of life positively	Low	Risk of bias – 1 Inconsistency – 1

### Mortality/suicide

The association between relational CoC and mortality/suicide was explored in three studies. A French observational study [37], based on a national insurance database included 14 515 patients who had visited a psychiatrist at least twice in 6 months and could be tracked over 3 years. Of these patients, 1 689 had a diagnosis of SMI. Exposure was measured by CoC-index [19] and outcome was mortality of all causes. The results for persons with bipolar disorder, major depressive disorder or schizophrenia showed, respectively, significant associations between CoC and all-cause mortality with adjusted hazard ratios between 0.84 and 0.87, p < 0.0001.

An American retrospective cohort study [44] included 144 045 patients within Veterans Administration diagnosed with SMI and co-occurring substance use disorder. Results were presented for each diagnostic subgroup. Exposure was defined as at least one diagnosis-related visit each quarter during a year. The outcome studied was mortality 12 and 24 months into the observation period. The group that had regular visits showed a 28% and 22% decrease in mortality at 12 and 24 months, respectively. A calculation of avoidable excess mortality showed that 656 and 984 lives respectively may have been saved by having higher CoC.

The relationship between CoC and suicide was explored in an American study based on four years’ data from a Veterans Administration register [34]. The sample included 121 933 patients with a diagnosis of major depression, bipolar disease, schizophrenia or PTSD. The authors calculated rate ratios for different categories of continuity in health service delivery. The authors operationalized the measure of continuity as the number of two-month periods with at least two visits in the first six months after discharge. One out of three analyses indicated results in favour of higher continuity whereas the other two did not. Thus, the interpretation of the data is complicated.

The overall result for the outcome mortality/suicide was *“Higher relational continuity of care for patients with serious mental illness may prevent premature mortality/suicide.”* The certainty of the evidence was assessed to be low, due to concerns of risk of bias due to confounding, inconsistency, and indirectness.

### Hospitalization

The association between relational CoC on risk of hospital readmission was explored in four studies. In a study from England^38^, 323 patients recently hospitalized for psychosis were followed for three years. CoC was assessed in eight dimensions, primarily measuring structural data like number of staff encountered in health care or length of gap between health visits. Outcomes were assessed as readmission and time to readmission. A few of the dimensions showed significant associations between CoC and readmission but the results as a whole did not show a uniform picture.

In a cross-national study of 7 302 patients from Belgium, England, Germany, Italy and Poland, diagnosed with a psychotic, mood or anxiety/somatization disorder, the significance of having one versus several psychiatrists was explored [36]. The design was described as a prospective natural experiment. The outcome was defined as readmission to hospital within a year from the index admission. The authors did not find any significant association between relational CoC and readmission to hospital.

A British study [42] focused on patients with SMI and their use of primary care. Relational CoC was measured by three indices (COC, UPC and SECON) and the outcomes chosen were emergency department (ED) visits, unplanned hospital admissions for SMI or ambulatory care-sensitive conditions (ACSC). Higher CoC was associated with a lower risk for ED presentations and ACSC, but not with risk for SMI admission.

In a study from the Netherlands [43] including 7 392 patients with schizophrenia, the focus was on the association between having continuous elective psychiatric care (1, 2 or 3 years) and four outcome measures: ED visits, psychiatric hospitalization, somatic care, and costs. The authors found negative associations between the exposure and the outcomes. The way of measuring continuity may, however, be questioned in relation to the aims of this review.

The overall result for the outcome hospitalization was *“Higher relational continuity of care for patients with severe mental illness may lower the risk of hospitalization”*. The certainty of the evidence was assessed to be very low, due to concerns regarding risk of bias, indirectness, and imprecision.

### Emergency department visits

Two studies presented above used ED visits as part of the outcome measure. One study [42] reported hazard ratios from several comparisons, both regarding ED visit frequency and a range of continuity measures. The hazard ratios were between 0.84 and 0.97 with five out of six results significant at the 0.05 level. The aggregated results showed that higher CoC was associated with lower frequency of ED visits, even though the magnitude of this effect was unclear. The other study [43] showed less frequent ED visits in the group with higher CoC.

A third study originated in Canada [38] investigated predictors of frequent ED visits for mental health reasons in 320 patients with mental health problems, the majority with a SMI diagnosis. The exposure was one of the so-called enabling factors which was operationalized as a regular source of care over a period of twelve months prior to inclusion. The results from a regression analysis were interpreted as a probable association between high CoC and less frequent ED visits. The choice of exposure may, however, be of questionable relevance for this review.

The overall result for the outcome ED visits was *“Higher relational continuity of care for patients with serious mental illness may reduce the risk of emergency room visits”*. The certainty of the evidence was assessed to be low, due to concerns regarding risk of bias and indirectness.

### Health care costs

Two studies investigated the effects of relational CoC on health costs. In an American study [32], 351 patients with schizophrenia were followed over a year’s time. Continuity was measured using three indices: COC (Continuity of Care), UPC (Usual Provider Continuity) and SECON (Sequential Continuity). One of the outcomes studied was payments for mental illness care and for total Medicaid care. The study showed a correlation between higher CoC and lower costs for both types of health care.

In a similar study from Canada [40], the authors investigated the relationship between CoC and costs for health and social care for 486 patients with psychosis or affective disorder with a 17-month follow-up. Continuity was measured with the instrument ACSS-MH. Most of the results showed statistically significant differences between separate cost categories for different levels of CoC. The differences were, however, not statistically significant for total health costs. In the Dutch study presented earlier [43], the authors describe an association between higher CoC and lower costs for mental health care. The authors estimate the effect size to range from moderate to high.

The overall result was formulated as: “*Higher relational continuity of care for patients with severe mental illness may lower health care costs”.* The certainty of the evidence was assessed to be very low, due to concerns with risk of bias, indirectness, and inconsistency.

### Symptoms and functioning

Three studies investigated the relation between CoC versus symptoms and functioning in patients with SMI [9, 31, 39]. A total of 5 832 patients with long-term psychosis, bipolar disorders or recurrent major depressions were included in the studies. In one study, the exposure was defined as the number of community “keyworkers” over a period of time [31]. In another study the exposure was defined as overall “experienced” continuity [9], whereas the third study used the instruments COC, UPC and SECON [28]. All of them investigated the effect of CoC on different clinical outcomes using linear regression analysis. The instruments were commonly used psychiatric scales like GAF (Global Assessment of Functioning), BPRS (Brief Psychiatric Rating Scale) and HoNOS (Health of the Nation Outcome Scale). Only one study presented results where the internal analyses are consistent [39], in this case showing an association between higher continuity with mental health teams and better outcome on scales measuring symptoms and/or functioning.

The overall result was formulated as: *“It is not possible to say whether relational continuity of care can improve symptoms and functioning in patients with serious mental illness.”* The certainty of the evidence was considered to be very low, due to concerns with indirectness, imprecision, and high risk of bias.

### Adherence to pharmacological treatment

In two large studies, the association between CoC and adherence to pharmacological treatment was studied. An Italian study included 11 797 patients with SMI [33]. Continuity was measured as at least one visit to mental health care each quarter in a year. Outcome was measured as adherence with pharmacological treatment based on prescription data. For patients with schizophrenia, there was a statistically significant relation between higher continuity and less risk for non-compliance with pharmacological treatment. No association was found for patients with bipolar disorder or major depression.

A similar study from the USA included 7 868 patients with schizophrenia [35]. Continuity of care was assessed as the number of prescribers. The outcome used was an index named medication possession ratio providing four categories of adherent behaviour: nonadherence, partial adherence, full adherence, and excess fillers. Patients with more prescribers were significantly more likely than patients with one prescriber to switch medications or fill prescriptions too soon.

The overall result was formulated as: “It is not possible to say whether relational continuity of care affects adherence to pharmacological treatment in patients with serious mental illness.” The certainty of the evidence was considered to be very low, due to concerns with indirectness, inconsistency, and high risk of bias in two of the three studies.

### Quality of life

Four studies investigated the association between CoC and quality of life (QOL). Two of these have already been presented under the heading symptoms and functioning [9], and under the heading costs [32]. A study from Canada included 486 patients with psychosis or affective disorder [29]. Relational CoC was measured with the instrument ACSS-MH and QOL was measured by the instrument EQ-5D (Euro Quality of Life – 5 Dimensions). A Norwegian study included 155 patients with schizophrenia, schizoaffective psychosis or bipolar disease [30]. Relational CoC was measured with the instrument CONTINU-UM and QOL was measured with the scale MANSA (The Manchester Short Assessment of Quality of Life). Three of the four studies used logistic regression. Two of them showed statistically significant relationships between CoC and QOL, whereas the other two did not find any significant associations.

The overall result was formulated as: *“Higher relational continuity of care for patients with serious mental illness may affect quality of life positively.”* The certainty of the evidence was considered to be low, due to high risk of bias and inconsistency.

## Discussion

The results in this systematic review indicate that higher relational continuity of care for patients with SMI may prevent premature deaths and suicide, may lower the number of ED visits, and may contribute to better quality of life compared to patients receiving lower levels of relational continuity of care. The certainty of the evidence was assessed as low, but the results show a high internal consistency with regard to the direction of the effects. The results were consistent across studies performed in different countries and health systems, across different methods used for measuring CoC, as well as across different analytic approaches. The certainty of the results related to risk of hospitalization, costs, symptoms and functioning, and adherence to drug treatment was too low for any reliable conclusions to be drawn. The general conclusion is nevertheless that higher relational continuity of care has been shown to have beneficial effects for patients with severe mental illness, and none of our results indicate an opposite relationship.

This systematic review was restricted to studies published in the English language from year 2000 and onward in international peer reviewed journals. This may include certain shortcomings in the review’s conclusions due to the risk of overlooking studies published in other languages or studies published before 2000. The study protocol included both controlled and observational studies. There were, however, no controlled studies available, which means that the review is entirely based on register or cohort studies, prospective or retrospective. This means that it is difficult to draw reliable conclusions regarding causality between exposure and outcome, which has affected the grading of the certainty of the results. It has therefore been of great importance for this review to consider in detail possible confounders and how these were dealt with by the different authors. Nevertheless, it cannot be ruled out that the results may be somewhat skewed due to residual confounding.

There was a considerable heterogeneity in the design of the studies. Nearly half of them were register studies which may contain some problems when it comes to the analytical choices made for both exposure and outcome. There were also some differences in how the exposure of continuity had been operationalized, measured, and classified in the analyses. This made it difficult to compare the results of the studies and furthermore to perform sound meta-analyses.

Many observational studies based on medical registers have a high quality with respect to coverage and variable definitions, and studies based on register data are most often large size studies on real everyday care. This review included 17 studies judged to be of satisfactory quality and relevant to the research question, and the total population included nearly 300 000 patients, which makes the conclusions reasonably reliable.

In most of the studies included in this review, illness severity and co-morbidity were potential confounders considered to a certain extent, but it cannot be ruled out that residual confounding remains. If all confounders relevant to illness severity and co-morbidity were also included, the effect results would probably be lowered which may be interpreted as a weakening of the association.

Serious mental illnesses are states of unhealth that can lead to major negative consequences in many fields of human life at the same time. The use of pharmaceutical agents is often needed but not always very successful. Some of these illnesses come and go over time, as in bipolar disorder, with quite varying needs in terms of health care, both with respect to quantity and quality.

These characteristics of SMI make it challenging to find ways within mental health care to help these patients in all different areas. SMI is a field where patients’ complex health care needs demand agile and flexible work over time. A relational continuity of care is probably one prerequisite for successful comprehensive health care that can be individually tailored towards each individual patient. This is especially important considering the continuous reorganizations of mental health care where less attention to continuity of care may imply worse clinical outcomes [39, 45].

It is clear that patients with SMI wish for better continuity of care than is the case today.^7^ This systematic review has shown that there is a potential for the development of even better relational continuity. It can therefore be seen as an ethical imperative that the health care system can offer good therapeutic relations that last over time with specific care givers to address the complex needs of patients with SMI.

The state of the art regarding the importance of CoC for patients with SMI gives hope but at the same time demands further development. Given the considerable heterogeneity in research methods, it would be very helpful for further research to establish better consensus with regard to more precise terms and better measures in the area. As patients with severe mental illness have multifaceted care needs and often have contacts with several caregivers, it would also be beneficial for future research to develop methods for studying the importance of relational continuity with teams. This was recently highlighted in a study where the use of care plans improved continuity and clinical outcomes [42].

Furthermore, since most studies so far have been designed as observational studies, future studies based on experimental designs should be attempted to heighten the current level of evidence. This is concluded in several earlier systematic reviews, e.g. in Puntis et al. [13], but is still relevant, as is the need for more consistent assessments of relational continuity of care.

## Conclusions

In summary, this systematic review provides some evidence that higher relational continuity of care for persons with SMI may prevent premature deaths and suicide, may lower the number of ED visits and may contribute to better quality of life compared to patients receiving lower levels of relational continuity of care. Even though the certainty of this evidence is assessed as being low, the concordance across studies is high. The finding that no studies indicate an inverse relationship between relational CoC and clinical outcomes is also notable. The heterogeneity of scientific methods is still a large problem in this area and should be addressed going forward through international cooperation in broad multinational studies. As noted in earlier studies we also conclude that there is a lack of good quality randomized controlled studies which in the future could add to the certainty of evidence in the area. Observational studies, especially register studies, however, have some advantages in comparison with experimental studies as they may target larger patient populations and outcomes of “real world” medical practice [46, 47].

Since the certainty of the combined evidence was assessed as low in this review, the evidence-base must be considered as remaining inconclusive. The finding that no studies indicate an inverse relationship between relational CoC and clinical outcomes is, however, notable and should be taken into account when organizing mental health care for patients with SMI. Since SMI is a chronic life-long condition, it is important to tailor the content of the treatment program individually, which includes raising awareness of the importance of relational continuity in healthcare provision.

### Electronic supplementary material

Below is the link to the electronic supplementary material.

**Supplementary Material 1:** Search strategies

**Supplementary Material 2:** Template for risk of bias assessment of studies regarding exposure

**Supplementary Material 3:** Assessment of risk of bias in relevant studies

**Supplementary Material 4:** Excluded studies

**Supplementary Material 5:** Detailed table of the included studies

## Data Availability

All data generated or analysed during this study are included in this published article and its supplementary information files.
