# Supplementary material for: Relational continuity may give better clinical outcomes in patients with serious mental illness – a systematic review
Source: BMC Psychiatry. 2023 Dec 18;23:952. doi: 10.1186/s12888-023-05440-1 (PMC10729558; doi:10.1186/s12888-023-05440-1)
Supplement: Supplementary file 1 — Supplementary Material 1: Search strategies [file 12888_2023_5440_MOESM1_ESM.docx]

Additional file 1 - Search strategies

Databases searched:

Cinahl

Clinicaltrials.gov

AHRQ

Cochrane library

CDR Databases via CRD

Embase

Epistemonikos

KSR Evidence

Medline

NICE evidence Search

Prospero

PsycINFO

Cinahl via EBSCO February 1, 2021

Title: Severe mental illness – relational continuity of care

| Search terms | | Items found |
| --- | --- | --- |
| Population: Severe mental illness | | |
|  | (MH "Depression+") OR (MH "Seasonal Affective Disorder") OR (MH "Psychotic Disorders+") OR (MH "Mental Disorders") | 285 279 |
|  | TI ( (serious* or severe*) NEAR/1 (mental* or psychiatric*) NEAR/1 (illness* or disorder* or disease*) ) OR AB ( (serious* or severe*) NEAR/1 (mental* or psychiatric*) NEAR/1 (illness* or disorder* or disease*) ) OR SU ( (serious* or severe*) NEAR/1 (mental* or psychiatric*) NEAR/1 (illness* or disorder* or disease*) ) OR TI ( (schizophren* or psychos?s* or psychotic* or bipolar or mania or manic or "manic-depress*" or dysthymi* or cyclothymi* or melancholi* or "seasonal affective disorder" or schizoaffective) ) OR AB ( (schizophren* or psychos?s* or psychotic* or bipolar or mania or manic or "manic-depress*" or dysthymi* or cyclothymi* or melancholi* or "seasonal affective disorder" or schizoaffective) ) OR SU ( (schizophren* or psychos?s* or psychotic* or bipolar or mania or manic or "manic-depress*" or dysthymi* or cyclothymi* or melancholi* or "seasonal affective disorder" or schizoaffective) ) OR TI ( (depression or "depressive disorder*") ) OR AB ( (depression or "depressive disorder*") ) OR SU ( (depression or "depressive disorder*") ) | 230 158 |
|  | *1-2 (OR)* | *350 611* |
| Intervention: Relational continuity of care | | |
|  | (MH "Continuity of Patient Care") | 15 095 |
|  | (continu*) N3 (physician* or practitioner* or doctor* or team* or provider* or nurs* or psychiatrist* or relation* or "cross-boundary*" or personal or interpersonal or care or healthcare) | 52 737 |
|  | ( (discontinu* or fragment* or inconsisten*) N3 (care or healthcare) ) OR ( ( (longitudinal*) N3 (care or healthcare) ) OR ( ("usual source" or "regular source") N3 (care or healthcare) ) OR ( (usual* or regular* or stabil*) N3 (physician* or practitioner* or doctor* or team* or provider* or nurs* or psychiatrist*) ) ) | 8 496 |
|  | *4-6(OR)* | *60 317* |
| Limits: publication year, language | | |
|  | Limiters - Published Date: 20000101-20211231; Language: Danish, English, Norwegian, Swedish |  |
| Combined sets | | |
|  | 3 AND 7 AND 8 | 2 780 |

The search result, usually found at the end of the documentation, forms the list of abstracts.

AB = Abstract

AU = Author

DE = Term from the thesaurus

MM = Major Concept

TI = Title

TX = All Text. Performs a keyword search of all the  database's searchable fields

ZC = Methodology Index

* = Truncation

“ “ = Citation Marks; searches for an exact phrase

*OBS!
Sökdokumentationen räknas som arbetsmaterial och får inte spridas utanför projektgruppen innan rapporten publiceras. Om sökstrategin i sin helhet används i andra sammanhang (t.e.x vid publicerande av artikel) bör man hänvisa till den publicerade sökdokumentationen på* [*www.sbu.se*](http://www.sbu.se)

**Clinicaltrials.gov 2021-02-04**

"continuity of care" or "care continuum" | Behaviors and Mental Disorders = 9

**AHRQ 2021-02-04**

”continuity” = 0

Cochrane Library via Wiley, February 3, 2021

Title: Severe mental illness – relational continuity of care

| Search terms | | | Items found | |
| --- | --- | --- | --- | --- |
| Population: Severe mental illness | | | | |
|  | [mh "Depressive Disorder"] | 12 298 | |  |
|  | [mh "Cyclothymic Disorder"] | 12 | |  |
|  | [mh ^"Mental Disorders"] | 3 750 | |  |
|  | [mh "Bipolar and Related Disorders"] | 2 686 | |  |
|  | [mh "Mood Disorders"] | 12 999 | |  |
|  | [mh "Schizophrenia Spectrum and Other Psychotic Disorders"] | 9 208 | |  |
|  | [mh "Psychotic Disorders"] | 3 008 | |  |
|  | [mh Schizophrenia] | 7 579 | |  |
|  | (((serious* or severe*) NEAR/1 (mental* or psychiatric*) NEAR/1 (illness* or disorder* or disease*))):ti,ab,kw OR (((schizophren* or psychos?s* or psychotic* or bipolar or mania or manic or manic* or depress* or dysthymi* or cyclothymi* or melancholi* or "seasonal affective disorder" or schizoaffective))):ti,ab,kw OR (depression or (depressive NEXT disorder*)):ti,ab,kw | 111 852 | |  |
|  | *1-9 (OR)* | *113 906* | |  |
| Exposure: Relational continuity of care | | | |  |
|  | [mh "Continuity of Patient Care"] | 617 | |  |
|  | (((continu*) NEAR/3 (physician* or practitioner* or doctor* or team* or provider* or nurs* or psychiatrist* or relation*)):ti,ab,kw OR (cross* NEXT (boundary* or personal or interpersonal or care or healthcare)):ti,ab,kw OR ((discontinu* or fragment* or inconsisten* OR longitudinal*) NEAR/3 (care or healthcare)):ti,ab,kw OR (("usual source" or "regular source") NEAR/3 (care or healthcare))):ti,ab,kw OR ((usual* or regular* or stabil*) NEAR/3 (physician* or practitioner* or doctor* or team* or provider* or nurs* or psychiatrist*)):ti,ab,kw | 3 477 | |  |
|  | *11-12 (OR)* | *4 053* | |  |
| Combined sets | | | |  |
|  | *10 AND 13* | *701*  *CDSR/15*  *Protocols/2*  *Central/686* | |  |

The search result, usually found at the end of the documentation, forms the list of abstracts.

:au = Author

MeSH = Term from the Medline controlled vocabulary, including terms found below this term in the MeSH hierarchy

this term only = Does not include terms found below this term in the MeSH hierarchy

:ti = title

:ab = abstract

:kw = keyword

* = Truncation

“ “ = Citation Marks; searches for an exact phrase

CDSR = Cochrane Database of Systematic Review

Protocols= Protocols for Cochrane reviews

CENTRAL = Cochrane Central Register of Controlled Trials, “trials”

CDR Databases via CRD, February 3, 2021 (DARE, NHS EED, HTA)

Title: Severe mental illness – relational continuity of care

| Search terms | | Items found |
| --- | --- | --- |
| Exposure: Relational continuity of care | | |
|  | MeSH DESCRIPTOR Continuity of Patient Care IN DARE,NHSEED,HTA  FROM 2000 TO 2020 | 142 |
|  | ( "continuity of care" OR "continuity of health care" OR "continuity of healthcare" OR "care continuity" OR "healthcare continuity" OR "team continuity" OR "provider continuity" OR "discontinuity of care" OR "discontinuity of health care" OR "discontinuity of healthcare" OR "fragmented care" OR "fragmented health care" OR "fragmented healthcare" OR "inconsistent care" OR "inconsistent health care" OR "inconsistent healthcare" OR "longitudinal care" OR "longitudinal health care" OR "longitudinal healthcare" OR "usual source of care" OR "usual source of health care" OR "usual source of healthcare" OR "regular source of care" OR "regular source of health care" OR "regular source of healthcare" OR "usual physician" OR "usual practitioner" or "usual doctor" OR "usual team" OR "usual provider" OR "usual nurse" OR "usual psychiatrist" OR "stable physician" OR "stable practitioner" or "stable doctor" OR "stable team" OR "stable provider" OR "stable nurse" OR "stable psychiatrist")  FROM 2000 TO 2021 | 65 |
| Combined sets | | |
|  | *1 OR 2* | *146* |

The search result, usually found at the end of the documentation, forms the list of abstracts.

:au = Author

MeSH = Term from the Medline controlled vocabulary, including terms found below this term in the MeSH hierarchy

this term only = Does not include terms found below this term in the MeSH hierarchy

:ti = title

:ab = abstract

:kw = keyword

* = Truncation

“ “ = Citation Marks; searches for an exact phrase

Embase via Elsevier, February 1, 2021

Title: Severe mental illness – relational continuity of care

| Search terms | | Items found |
| --- | --- | --- |
| Population: Severe mental disorders | | |
|  | 'depression'/exp | 518 229 |
|  | 'mood disorder'/exp OR 'psychosis'/exp OR 'schizophrenia spectrum disorder'/exp | 789 531 |
|  | 'mental disease'/de | 255 228 |
|  | ((serious* OR severe*) NEAR/1 (mental* OR psychiatric*) NEAR/1 (illness* OR disorder* OR disease*)):ab,ti | 14 804 |
|  | schizophren*:ab,ti OR psychos?s*:ab,ti OR psychotic*:ab,ti OR bipolar:ab,ti OR mania:ab,ti OR manic:ab,ti OR 'manic-depress*':ab,ti OR dysthymi*:ab,ti OR cyclothymi*:ab,ti OR melancholi*:ab,ti OR 'seasonal affective disorder':ab,ti OR schizoaffective:ab,ti | 328 497 |
|  | depression OR 'depressive disorder*':ab,ti | 719 473 |
|  | *1-6 (OR)* | *1 245 504* |
| Intervention: Continuity of care | | |
|  | 'continuity effect'/exp | 23 |
|  | (continu* NEAR/3 (physician* OR practitioner* OR doctor* OR team* OR provider* OR nurs* OR psychiatrist* OR relation* OR 'cross-boundary*' OR personal OR interpersonal OR care OR healthcare)):ab,ti | 46 541 |
|  | ((discontinu* OR fragment* OR inconsisten*) NEAR/3 (care OR healthcare)):ab,ti | 3 818 |
|  | (longitudinal* NEAR/3 (care OR healthcare)):ab,ti | 1 958 |
|  | (('usual source' OR 'regular source') NEAR/3 (care OR healthcare)):ab,ti | 1 470 |
|  | ((usual* OR regular* OR stabil*) NEAR/3 (physician* OR practitioner* OR doctor* OR team* OR provider* OR nurs* OR psychiatrist*)):ab,ti | 9 154 |
|  | *8-13 (OR)* | *61 644* |
| Limits: language, publication year, publication type | | |
|  | [danish]/lim OR [english]/lim OR [norwegian]/lim OR [swedish]/lim |  |
|  | [2000-2021]/py |  |
|  | ([conference abstract]/lim OR [conference paper]/lim OR [conference review]/lim OR [data papers]/lim) |  |
| Combined sets | | |
|  | *(7 AND 14 AND 15 AND 16) NOT 17* | *2 708* |

The search result, usually found at the end of the documentation, forms the list of abstracts.

*OBS!
Sökdokumentationen räknas som arbetsmaterial och får inte spridas utanför projektgruppen innan rapporten publiceras. Om sökstrategin i sin helhet används i andra sammanhang (t.e.x vid publicerande av artikel) bör man hänvisa till den publicerade sökdokumentationen på* [*www.sbu.se*](http://www.sbu.se)

*OBS!*

*Sökdokumentationen räknas som arbetsmaterial och får inte spridas utanför projektgruppen innan rapporten publiceras. Om sökstrategin i sin helhet används i andra sammanhang (t.e.x vid publicerande av artikel) bör man hänvisa till den publicerade sökdokumentationen på* [*www.sbu.se*](http://www.sbu.se)

/de= Term from the EMTREE controlled vocabulary

/exp= Includes terms found below this term in the EMTREE hierarchy

/mj = Major Topic

:ab = Abstract

:au = Author

:ti = Article Title

:ti:ab = Title or abstract

* = Truncation

“ “ = Citation Marks; searches for an exact phrase

Epistemonikos via Epistemonikos, February 3, 2021

Title: Severe mental illness – relational continuity of care

| Search terms | | Items found | |  |  |
| --- | --- | --- | --- | --- | --- |
| Exposure: Relational continuity of care | | | |  |  |
|  | (title:(continuity OR "care continuum" OR "healthcare continuum" OR "health care continuum" OR "longitudinal care" OR "longitudinal healthcare" OR "discontinuity of care" OR "fragmented care" OR "care fragmentation" OR "fragmentation of care" OR "inconsistent care" OR "discontinuity of healthcare" OR "fragmented healthcare" OR "healthcare fragmentation" OR "fragmentation of healthcare" OR "inconsistent healthcare" OR "usual doctor*" OR "usual provider*" OR "usual physician*" OR "usual practitioner*" OR "usual nurse*" OR "usual team*" OR "usual psychiatrist*" OR "usual source of care" OR "usual source of healthcare" OR "regular source of care" OR "regular source of healthcare" OR "provider stability" OR "care stability" OR "healthcare stability" OR "regular doctor*" OR "regular provider*" OR "regular physician*" OR "regular practitioner*" OR "regular nurse*" OR "regular team*" OR "regular psychiatrist*") OR abstract:(continuity OR "care continuum" OR "healthcare continuum" OR "health care continuum" OR "longitudinal care" OR "longitudinal healthcare" OR "discontinuity of care" OR "fragmented care" OR "care fragmentation" OR "fragmentation of care" OR "inconsistent care" OR "discontinuity of healthcare" OR "fragmented healthcare" OR "healthcare fragmentation" OR "fragmentation of healthcare" OR "inconsistent healthcare" OR "usual doctor*" OR "usual provider*" OR "usual physician*" OR "usual practitioner*" OR "usual nurse*" OR "usual team*" OR "usual psychiatrist*" OR "usual source of care" OR "usual source of healthcare" OR "regular source of care" OR "regular source of healthcare" OR "provider stability" OR "care stability" OR "healthcare stability" OR "regular doctor*" OR "regular provider*" OR "regular physician*" OR "regular practitioner*" OR "regular nurse*" OR "regular team*" OR "regular psychiatrist*")) | | 2 567 | | |
| Population:Severe mental disorders | | | | |  |
|  | (title:("serious mental illness" OR "serious mental disorder" OR "serious mental disease" OR "severe mental illness" OR "severe mental disorder" OR "severe mental disease" OR "serious psychiatric illness" OR "serious psychiatric disorder" OR "serious psychiatric disease" OR "severe psychiatric illness" OR "severe psychiatric disorder" OR "severe psychiatric disease" or) OR abstract:("serious mental illness" OR "serious mental disorder" OR "serious mental disease" OR "severe mental illness" OR "severe mental disorder" OR "severe mental disease" OR "serious psychiatric illness" OR "serious psychiatric disorder" OR "serious psychiatric disease" OR "severe psychiatric illness" OR "severe psychiatric disorder" OR "severe psychiatric disease" or)) OR (title:(schizophren* OR psychosis OR psychoses OR psychotic* OR bipolar OR mania OR manic OR "manic-depress*" OR dysthymi* OR cyclothymi* OR melancholi* OR "seasonal affective disorder" OR schizoaffective) OR abstract:(schizophren* OR psychosis OR psychoses OR psychotic* OR bipolar OR mania OR manic OR "manic-depress*" OR dysthymi* OR cyclothymi* OR melancholi* OR "seasonal affective disorder" OR schizoaffective)) OR (title:(depression OR "depressive disorder*") OR abstract:(depression OR "depressive disorder*")) | | 502 093 | | |
| Limits: publication year | | | | |  |
|  | 21002000-2021 | |  | | |
| Combined sets | | | | |  |
|  | *1 A21 AND 2 AND 3* | | *1 21 262* | | |

All text= title, abstract, bottom line, Risk of Bias Assessment, Results, Details

KSR Evidence via KSR, February 3, 2021

Title: Severe mental illness – relational continuity of care

| Search terms | | Items found |
| --- | --- | --- |
| Exposure: Relational continuity of care | | |
|  | continuity OR "care continuum" OR "healthcare continuum" OR "health care continuum" in All text | 409 |
|  | "longitudinal care" or "longitudinal healthcare" in All text | 1 |
|  | "discontinuity of care" or "fragmented care" or "care fragmentation" or "fragmentation of care" or "inconsistent care" or "discontinuity of healthcare" or "fragmented healthcare" or "healthcare fragmentation" or "fragmentation of healthcare" or "inconsistent healthcare" in All text | 18 |
|  | "usual doctor*" or "usual provider*" or "usual physician*" or "usual practitioner*" or "usual nurse*" or "usual team*" or "usual psychiatrist*" in All text | 5 |
|  | "usual source of care" or "usual source of healthcare" or "regular source of care" or "regular source of healthcare" or "provider stability" or "care stability" or "healthcare stability" in All text | 2 |
|  | "regular doctor*" or "regular provider*" or "regular physician*" or "regular practitioner*" or "regular nurse*" or "regular team*" or "regular psychiatrist*" in All text | 6 |
|  | *1-6 (OR)* | *437* |

All text= title, abstract, bottom line, Risk of Bias Assessment, Results, Details

Medline via OvidSP, February 1, 2021

Title: Severe mental illness – relational continuity of care

| Search terms | | Items found | |
| --- | --- | --- | --- |
| Population: Severe mental disorders | | | |
|  | depressive disorder/ or depressive disorder, major/ or depressive disorder, treatment-resistant/ or dysthymic disorder/ or seasonal affective disorder/ or cyclothymic disorder/ or mental disorders/ or exp "bipolar and related disorders"/ or exp mood disorders/ or exp "schizophrenia spectrum and other psychotic disorders"/ or exp psychotic disorders/ or exp schizophrenia/ | | 429 357 |
|  | ((serious* or severe*) adj (mental* or psychiatric*) adj (illness* or disorder* or disease*)).ab,kf,ti. | | 11 564 |
|  | (schizophren* or psychos#s* or psychotic* or bipolar or mania or manic or manic-depress* or dysthymi* or cyclothymi* or melancholi* or "seasonal affective disorder" or schizoaffective).ab,kf,ti. | | 242 631 |
|  | (depression or "depressive disorder*").ab,kf,ti. | | 368 489 |
|  | *1-4 (OR)* | | *762 068* |
| Exposure: Relational continuity of care | | | |
|  | "Continuity of Patient Care"/ | | 19 008 |
|  | (continu* adj3 (physician* or practitioner* or doctor* or team* or provider* or nurs* or psychiatrist* or relation* or "cross-boundary*" or personal or interpersonal or care or healthcare)).ti,ab,kf. | | 35 033 |
|  | ((discontinu* or fragment* or inconsisten*) adj3 (care or healthcare)).ti,ab,kf. | | 2 646 |
|  | (longitudinal* adj3 (care or healthcare)).ti,ab,kf. | | 298 |
|  | (("usual source" or "regular source") adj3 (care or healthcare)).ti,ab,kf. | | 1 288 |
|  | ((usual* or regular* or stabil*) adj3 (physician* or practitioner* or doctor* or team* or provider* or nurs* or psychiatrist*)).ti,ab,kf. | | 6 396 |
|  | *6-11 (OR)* | | *59 512* |
| Limits: publication date, language | | | |
|  | yr="2000 -Current" | |  |
|  | *danish or english or norwegian or swedish* | |  |
| Combined sets | | | |
|  | 5 AND 12 AND 13 AND 14 | | 2 610 |

The search result, usually found at the end of the documentation, forms the list of abstracts.

.ab. =Abstract

.ab,ti. = Abstract or title

.af.= All fields

Exp= Term from the Medline controlled vocabulary, including terms found below this term in the MeSH hierarchy

.sh.= Term from the Medline controlled vocabulary

.ti. = Title

/ = Term from the Medline controlled vocabulary, but does not include terms found below this term in the MeSH hierarchy

* = Focus (if found in front of a MeSH-term)

* or $= Truncation (if found at the end of a free text term)

.mp=text, heading word, subject area node, title

NICE Evidence Search via NICE, February 4, 2021

Title: Severe mental illness – relational continuity of care

| Search terms | | Items found |
| --- | --- | --- |
| Exposure: Relational continuity of care | | |
|  | “continuity of care”  Systematic Reviews, Health Technology Assessment  20000101-20210204 | **213** |

The search result, usually found at the end of the documentation, forms the list of abstracts.

[MeSH] = Term from the Medline controlled vocabulary, including terms found below this term in the MeSH hierarchy

[MeSH:NoExp] = Does not include terms found below this term in the MeSH hierarchy

[MAJR] = MeSH Major Topic

[TIAB] = Title or abstract

[TI] = Title

[AU] = Author

[OT]= Other term

[TW] = Text Word

Systematic[SB] = Filter for retrieving systematic reviews

* = Truncation

PROSPERO via CRD 4 February 2021 (DARE, NHS EED, HTA)

Title: Severe mental illness – relational continuity of care

| Search terms | | Items found |
| --- | --- | --- |
| Exposure: Relational continuity of care | | |
|  | MeSH DESCRIPTOR Continuity of Patient Care | 1 |
|  | "continuity of care" OR "continuity of health care" OR "continuity of healthcare" OR "care continuity" OR "healthcare continuity" OR "team continuity" OR "provider continuity" OR "discontinuity of care" OR "discontinuity of health care" OR "discontinuity of healthcare" OR "fragmented care" OR "fragmented health care" OR "fragmented healthcare" OR "inconsistent care" OR "inconsistent health care" OR "inconsistent healthcare" OR "longitudinal care" OR "longitudinal health care" OR "longitudinal healthcare" OR "usual source of care" OR "usual source of health care" OR "usual source of healthcare" OR "regular source of care" OR "regular source of health care" OR "regular source of healthcare" OR "usual physician" OR "usual practitioner" or "usual doctor" OR "usual team" OR "usual provider" OR "usual nurse" OR "usual psychiatrist" OR "stable physician" OR "stable practitioner" or "stable doctor" OR "stable team" OR "stable provider" OR "stable nurse" OR "stable psychiatrist" | 190 |
| Combined sets | | |
|  | *1 OR 2* | *1190* |

The search result, usually found at the end of the documentation, forms the list of abstracts.

:au = Author

MeSH = Term from the Medline controlled vocabulary, including terms found below this term in the MeSH hierarchy

this term only = Does not include terms found below this term in the MeSH hierarchy

:ti = title

:ab = abstract

:kw = keyword

* = Truncation

“ “ = Citation Marks; searches for an exact phrase

PsycINFO via EBSCO 1 February 2021

Title: Severe mental illness – relational continuity of care

| Search terms | | Items found |
| --- | --- | --- |
| Population: Severe mental disorders | | |
|  | (((((DE "Major Depression" OR DE "Anaclitic Depression" OR DE "Dysthymic Disorder" OR DE "Endogenous Depression" OR DE "Late Life Depression" OR DE "Postpartum Depression" OR DE "Reactive Depression" OR DE "Recurrent Depression" OR DE "Treatment Resistant Depression") OR (DE "Bipolar Disorder" OR DE "Bipolar I Disorder" OR DE "Bipolar II Disorder" OR DE "Cyclothymic Disorder" OR DE "Mania")) OR (DE "Psychosis" OR DE "Acute Psychosis" OR DE "Affective Psychosis" OR DE "Alcoholic Psychosis" OR DE "Capgras Syndrome" OR DE "Childhood Psychosis" OR DE "Chronic Psychosis" OR DE "Experimental Psychosis" OR DE "Hallucinosis" OR DE "Paranoia (Psychosis)" OR DE "Postpartum Psychosis" OR DE "Reactive Psychosis" OR DE "Schizophrenia" OR DE "Senile Psychosis" OR DE "Toxic Psychoses")) OR (DE "Schizophrenia" OR DE "Acute Schizophrenia" OR DE "Catatonic Schizophrenia" OR DE "Childhood Schizophrenia" OR DE "Paranoid Schizophrenia" OR DE "Process Schizophrenia" OR DE "Schizoaffective Disorder" OR DE "Schizophrenia (Disorganized Type)" OR DE "Schizophreniform Disorder" OR DE "Undifferentiated Schizophrenia")) OR (DE "Paranoia (Psychosis)" OR DE "Folie A Deux")) OR (DE "Hallucinosis" OR DE "Alcoholic Hallucinosis") | 277 070 |
|  | TI ( (serious* or severe*) NEAR/1 (mental* or psychiatric*) NEAR/1 (illness* or disorder* or disease*) ) OR AB ( (serious* or severe*) NEAR/1 (mental* or psychiatric*) NEAR/1 (illness* or disorder* or disease*) ) OR SU ( (serious* or severe*) NEAR/1 (mental* or psychiatric*) NEAR/1 (illness* or disorder* or disease*) ) OR TI ( (schizophren* or psychos?s* or psychotic* or bipolar or mania or manic or "manic-depress*" or dysthymi* or cyclothymi* or melancholi* or "seasonal affective disorder" or schizoaffective) ) OR AB ( (schizophren* or psychos?s* or psychotic* or bipolar or mania or manic or "manic-depress*" or dysthymi* or cyclothymi* or melancholi* or "seasonal affective disorder" or schizoaffective) ) OR SU ( (schizophren* or psychos?s* or psychotic* or bipolar or mania or manic or "manic-depress*" or dysthymi* or cyclothymi* or melancholi* or "seasonal affective disorder" or schizoaffective) ) OR TI ( (depression or "depressive disorder*") ) OR AB ( (depression or "depressive disorder*") ) OR SU ( (depression or "depressive disorder*") ) | 481 783 |
|  | *1-2 (OR)* | *482 160* |
| Intervention: Relational continuity of care | | |
|  | DE "Continuum of Care" | 1 887 |
|  | (continu*) N3 (physician* or practitioner* or doctor* or team* or provider* or nurs* or psychiatrist* or relation* or "cross-boundary*" or personal or interpersonal or care or healthcare) | 19 276 |
|  | (discontinu* or fragment* or inconsisten*) N3 (care or healthcare) | 979 |
|  | ( (longitudinal*) N3 (care or healthcare) ) OR ( ("usual source" or "regular source") N3 (care or healthcare) ) OR ( (usual* or regular* or stabil*) N3 (physician* or practitioner* or doctor* or team* or provider* or nurs* or psychiatrist*) ) | 3 615 |
|  | *4-7 (OR)* | *23 470* |
| Limits: Publication year, language | | |
|  | Limiters - Publication Year: 2000-2021; Language: Danish, English, Norwegian, Swedish |  |
| Combined sets | | |
|  | 3 AND 8 AND 9 | 1 677 |

The search result, usually found at the end of the documentation, forms the list of abstracts.

AB = Abstract

AU = Author

DE = Term from the thesaurus

MM = Major Concept

TI = Title

TX = All Text. Performs a keyword search of all the  database's searchable fields

ZC = Methodology Index

* = Truncation

“ “ = Citation Marks; searches for an exact phrase

Scopus via Elsevier, February 2, 2021

Title: Severe mental illness – relational continuity of care

| Search terms | | Items found |
| --- | --- | --- |
| Population: Severe mental disorders | | |
|  | ( TITLE-ABS-KEY ( ( ( serious* OR severe* ) W/1 ( mental* OR psychiatric* ) W/1 ( illness* OR disorder* OR disease* ) ) ) OR TITLE-ABS-KEY ( ( schizophren* OR psychosis* OR psychoses* OR psychotic* OR bipolar OR mania OR manic OR manic-depress* OR dysthymi* OR cyclothymi* OR melancholi* OR "seasonal affective disorder" OR schizoaffective ) ) OR TITLE-ABS-KEY ( depression OR "depressive disorder*" ) ) | 1 132 961 |
| Exposure: Relational continuity of care | | |
|  | ( TITLE-ABS-KEY ( ( ( continu* ) W/3 ( physician* OR practitioner* OR doctor* OR team* OR provider* OR nurs* OR psychiatrist* OR relation* OR "cross-boundary*" OR personal OR interpersonal OR care OR healthcare ) ) ) ) OR ( TITLE-ABS-KEY ( ( ( discontinu* OR fragment* OR inconsisten* ) W/3 ( care OR healthcare ) ) ) ) OR ( TITLE-ABS-KEY ( ( ( longitudinal* ) W/3 ( care OR healthcare ) ) ) ) OR ( TITLE-ABS-KEY ( ( ( "usual source" OR "regular source" ) W/3 ( care OR healthcare ) ) ) ) OR ( TITLE-ABS-KEY ( ( ( usual* OR regular* OR stabil* ) W/3 ( physician* OR practitioner* OR doctor* OR team* OR provider* OR nurs* OR psychiatrist* ) ) ) ) | 116 356 |
| Limits: publication year, language | | |
|  | ( LIMIT-TO ( PUBYEAR , 2021 ) OR LIMIT-TO ( PUBYEAR , 2020 ) OR LIMIT-TO ( PUBYEAR , 2019 ) OR LIMIT-TO ( PUBYEAR , 2018 ) OR LIMIT-TO ( PUBYEAR , 2017 ) OR LIMIT-TO ( PUBYEAR , 2016 ) OR LIMIT-TO ( PUBYEAR , 2015 ) OR LIMIT-TO ( PUBYEAR , 2014 ) OR LIMIT-TO ( PUBYEAR , 2013 ) OR LIMIT-TO ( PUBYEAR , 2012 ) OR LIMIT-TO ( PUBYEAR , 2011 ) OR LIMIT-TO ( PUBYEAR , 2010 ) OR LIMIT-TO ( PUBYEAR , 2009 ) OR LIMIT-TO ( PUBYEAR , 2008 ) OR LIMIT-TO ( PUBYEAR , 2007 ) OR LIMIT-TO ( PUBYEAR , 2006 ) OR LIMIT-TO ( PUBYEAR , 2005 ) OR LIMIT-TO ( PUBYEAR , 2004 ) OR LIMIT-TO ( PUBYEAR , 2003 ) OR LIMIT-TO ( PUBYEAR , 2002 ) OR LIMIT-TO ( PUBYEAR , 2001 ) OR LIMIT-TO ( PUBYEAR , 2000 ) ) AND ( LIMIT-TO ( LANGUAGE , "English" ) OR LIMIT-TO ( LANGUAGE , "Swedish" ) OR LIMIT-TO ( LANGUAGE , "Norwegian" ) ) |  |
| Combined sets | | |
|  | 1 AND 2 AND 3 | 3 363 |

The search result, usually found at the end of the documentation, forms the list of abstracts.

TITLE-ABS-KEY  = Title or abstract or keywords
**ALL** = All fields
**W/n** = "within". The terms in the search must be within a specified number of terms (n) in any order.
***** = Truncation

**“ “** = Citation Marks; searches for an exact phrase

LIMIT-TO ( SRCTYPE ,  "j"  = Limit to source type journal
LIMIT-TO ( DOCTYPE ,  "ar"  = Limit to document type article
LIMIT-TO ( DOCTYPE ,  "re"   = Limit to document type review

*OBS!
Sökdokumentationen räknas som arbetsmaterial och får inte spridas utanför projektgruppen innan rapporten publiceras. Om sökstrategin i sin helhet används i andra sammanhang (t.e.x vid publicerande av artikel) bör man hänvisa till den publicerade sökdokumentationen på* [*www.sbu.se*](http://www.sbu.se)
