# Supplementary material for: Relational continuity may give better clinical outcomes in patients with serious mental illness – a systematic review
Source: BMC Psychiatry. 2023 Dec 18;23:952. doi: 10.1186/s12888-023-05440-1 (PMC10729558; doi:10.1186/s12888-023-05440-1)
Supplement: Supplementary file 2 — Supplementary Material 2: Template for risk of bias assessment of studies regarding exposure [file 12888_2023_5440_MOESM2_ESM.docx]

**Additional file 2**

*OBS!
Sökdokumentationen räknas som arbetsmaterial och får inte spridas utanför projektgruppen innan rapporten publiceras. Om sökstrategin i sin helhet används i andra sammanhang (t.e.x vid publicerande av artikel) bör man hänvisa till den publicerade sökdokumentationen på* [*www.sbu.se*](http://www.sbu.se)

# Template for risk of bias assessment of studies regarding exposure

Reference (author, year): ______________________________________________

Outcome: ___________________________________________________________

Assessor: _________________________________________________________

| To consider before assessment:Has the project chosen to use adjusted or unadjusted measures? |
| --- |

# **1. Bias due to confounding**

| Risk of bias due to confounding: | Low  Moderate  High   Unacceptable  | | | | |
| --- | --- | --- | --- | --- | --- |
|  | **Yes** | **Probably yes** | **Probably no** | **No** | **Information missing** |
| 1.A Have important confounders been considered*? | ❑ | ❑ | ❑ | ❑ | (Option not available for this question) |
| 1.B Were confounders measured using valid and reliable methods? | ❑ | ❑ | ❑ | ❑ | (Option not available for this question) |

**Possible confounders that have been discussed:*

- *Degree of morbidity*
- *Age*
- *Attitude (those with more knowledge can manage on their own)*
- *Expectancy of continuity, e.g., online doctors*
- *Social support, relatives, marital status*
- *Distance from health centre*
- *Socioeconomics and/or education, ability to pay*

**Motivation for assessment (if relevant):**

# **2. Bias due to exposure**

| Risk of bias due to exposure | Low  Moderate  High   Unacceptable  | | | | |
| --- | --- | --- | --- | --- | --- |
|  | **Yes** | **Probably yes** | **Probably no** | **No** | **Information missing** |
| 2.A Is the exposure relevant and well defined considering the authors’ research question? | ❑ | ❑ | ❑ | ❑ | ❑ |
| 2.B Is the exposure variable measured using valid and reliable methods? | ❑ | ❑ | ❑ | ❑ | ❑ |
| 2.C Is there a risk of misclassification/wrong assessment in relation to the project’s research question? | ❑ | ❑ | ❑ | ❑ | ❑ |
| 2.D If comparing between groups: were groups recruited in different ways so that there is a risk of bias due to differences in exposure? | ❑ | ❑ | ❑ | ❑ | ❑ |

Footnote: questions 2.C and 2.D should only be answered if they are deemed relevant

**Motivation for assessment (if relevant):**

# **3. Bias due to drop-out**

| Risk of bias due to drop-out | Low  Moderate  High   Unacceptable  | | | | |
| --- | --- | --- | --- | --- | --- |
|  | **Yes** | **Probably yes** | **Probably no** | **No** | **Information missing** |
| 3.A Is there a significant drop-out after inclusion? | ❑ | ❑ | ❑ | ❑ | ❑ |
| 3.B Has drop-out been handled in a satisfactory manner? | ❑ | ❑ | ❑ | ❑ | ❑ |

Footnote: if question 3.A is answered with no, question 3.B need not be answered

**Motivation for assessment (if relevant):**

# **4. Measurement and analysis of outcome measure**

| Risk of bias due to measurement and analysis of outcome measure | Low  Moderate  High   Unacceptable  | | | | |
| --- | --- | --- | --- | --- | --- |
|  | **Yes** | **Probably yes** | **Probably no** | **No** | **Information missing** |
| 4.A Is the outcome well defined considering the study’s research question? | ❑ | ❑ | ❑ | ❑ | ❑ |
| 4.B Is the outcome measured using valid and reliable methods? | ❑ | ❑ | ❑ | ❑ | ❑ |
| 4.C Is the outcome measured at reasonable time points? | ❑ | ❑ | ❑ | ❑ | ❑ |
| 4.D Has the study applied suitable statistical methods to analyse the data? | ❑ | ❑ | ❑ | ❑ | ❑ |

**Motivation for assessment (if relevant):**

# **5.Bias due to reporting**

| Risk of bias due to reporting | Low ❑ Moderate ❑ High   Unacceptable  | | | | |
| --- | --- | --- | --- | --- | --- |
|  | **Yes** | **Probably yes** | **Probably no** | **No** | **Information missing** |
| - 5.A Has the outcome measure been reported in an adequate manner considering the study’s research question? | ❑ | ❑ | ❑ | ❑ | ❑ |
| - 5.B Do the authors report that there is a published study protocol or analysis plan? | ❑ | ❑ | ❑ | ❑ | ❑ |

**Motivation for assessment (if relevant):**

# **6. Bias due to conflicts of interest**

| Bias due to conflicts of interest | Low  Moderate  High   Unacceptable  | | | | |
| --- | --- | --- | --- | --- | --- |
|  | **Yes** | **Probably yes** | **Probably no** | **No** | **Information missing** |
| - 6.A Is there a low risk that the study’s results have been influenced by conflicts of interest, based on the affiliations and competing interests that the authors have declared? | ❑ | ❑ | ❑ | ❑ | ❑ |
| - 6.B Is there a low risk that the study’s results were influenced by a funding body with economic interests in the results? | ❑ | ❑ | ❑ | ❑ | ❑ |

**Motivation for assessment (if relevant):**

| Summary of risk of bias per domain and overall | | | | |
| --- | --- | --- | --- | --- |
| Risk of… | **Low** | **Moderate** | **High** | **Unacceptable** |
| 1. Bias due to confounding | ❑ | ❑ | ❑ | ❑ |
| 1. Bias due to exposure | ❑ | ❑ | ❑ | ❑ |
| 1. Bias due to drop-out | ❑ | ❑ | ❑ | ❑ |
| 1. Bias due to measurement and analysis of outcome | ❑ | ❑ | ❑ | ❑ |
| 1. Bias due to reporting | ❑ | ❑ | ❑ | ❑ |
| 1. Bias due to conflicts of interest | ❑ | ❑ | ❑ | ❑ |
|  |  |  |  |  |
| - Overall risk of bias for outcome | ❑ | ❑ | ❑ | ❑ |

**Motivation for assessment (if relevant):**
