# Supplementary material for: Relational continuity may give better clinical outcomes in patients with serious mental illness – a systematic review
Source: BMC Psychiatry. 2023 Dec 18;23:952. doi: 10.1186/s12888-023-05440-1 (PMC10729558; doi:10.1186/s12888-023-05440-1)
Supplement: Supplementary file 3 — Supplementary Material 3: Assessment of risk of bias in relevant studies [file 12888_2023_5440_MOESM3_ESM.docx]

# **Additional file 3 - Assessment of risk of bias in relevant studies**

| **Study** | **Confounding** | **Exposure** | **Drop-out** | **Measurement and analysis** | **Reporting** | **Conflicts of interest** | **Overall RoB** |
| --- | --- | --- | --- | --- | --- | --- | --- |
| Adair et al.* [1] | Moderate | Low | Moderate | Moderate | Moderate | Low | High |
| Adnanes et al. [2] | Moderate | Low | Moderate | Low | Low | Low | Moderate |
| Bindman et al. [3] | Moderate | Low | Moderate | Moderate | Low | Low | High |
| Catty et al. [4] | High | Moderate | High | Moderate | Moderate | Low | High |
| Chien et al. [5] | Moderate | Low | Low | Moderate | Low | Low | Moderate |
| Conti et al. [6] | Moderate | Low | Low | Low | Low | Low | Moderate |
| Desai et.al.[7] | Moderate | Moderate | Low | Low | Low | Low | Moderate |
| Farley et al. [8] | Moderate | Low | Moderate | Low | Low | Low | Moderate |
| Fleury et al. [9] | High | High | Moderate | High | Moderate | Low | Unacceptable |
| Giacco et al. [10] | Moderate | Moderate | Low | Low | Low | Low | Moderate |
| Green et al. [11] | High | Moderate | Moderate | High | Moderate | Low | Unacceptable |
| Hoertel et al. [12] | Moderate | Low | Low | Low | Low | Low | High |
| Kaltsidis et al. [13] | Moderate | Moderate | Low | Low | Low | Low | Moderate |
| MacDonald et al. [14] | Moderate | Moderate | Low | Moderate | Low | Low | High |
| Mitton et al.* [15] | Moderate | Low | Low | Low | Low | Low | Moderate |
| Puntis et al. [16] | Moderate | Low | Low | Low | Low | Low | Moderate |
| Ride et al. [17] | Moderate | Low | Moderate | Low | Low | Low | Moderate |
| van der Lee et al. [18] | High | Low | Moderate | Low | Low | Low | High |
| Watkins et al. [19] | Moderate | Low | Low | Low | Low | Low | Moderate |

*The articles are based on the same study and report different outcome measures.

##

## ***References***

1. Adair CE, McDougall GM, Mitton CR, Joyce AS, Wild TC, Gordon A, et al. Continuity of care and health outcomes among persons with severe mental illness. Psychiatric Services. 2005;56(9):1061-9. Available from: <https://doi.org/10.1176/appi.ps.56.9.1061>.
2. Adnanes M, Kalseth J, Ose SO, Ruud T, Rugkasa J, Puntis S. Quality of life and service satisfaction in outpatients with severe or non-severe mental illness diagnoses. Quality of Life Research. 2019;28(3):713-24. Available from: <https://doi.org/10.1007/s11136-018-2039-5>.
3. Bindman J, Johnson S, Szmukler G, Wright S, Kuipers E, Thornicroft G, et al. Continuity of care clinical outcome: A prospective cohort study. Social Psychiatry and Psychiatric Epidemiology. 2000;35(6):242-7. Available from: <https://doi.org/10.1007/s001270050234>.
4. Catty J, White S, Clement S, Cowan N, Geyer C, Harvey K, et al. Continuity of care for people with psychotic illness: its relationship to clinical and social functioning. International Journal of Social Psychiatry. 2013;59(1):5-17. Available from: <https://doi.org/10.1177/0020764011421440>.
5. Chien CF, Steinwachs DM, Lehman A, Fahey M, Skinner EA. Provider continuity and outcomes of care for persons with schizophrenia. Mental Health Services Research. 2000;2(4):201-11. Available from: <https://doi.org/10.1023/A:1010160419561>.
6. Conti V, Lora A, Cipriani A, Fortino I, Merlino L, Barbui C. Persistence with pharmacological treatment in the specialist mental healthcare of patients with severe mental disorders. European Journal of Clinical Pharmacology. 2012;68(12):1647-55. Available from: <https://doi.org/10.1007/s00228-012-1298-2>.
7. Desai RA, Dausey DJ, Rosenheck RA. Mental health service delivery and suicide risk: the role of individual patient and facility factors. American Journal of Psychiatry. 2005;162(2):311-8. Available from: <https://doi.org/10.1176/appi.ajp.162.2.311>.
8. Farley JF, Wang CC, Hansen RA, Voils CI, Maciejewski ML. Continuity of antipsychotic medication management for Medicaid patients with schizophrenia. Psychiatric Services. 2011;62(7):747-52. Available from: <https://doi.org/10.1176/ps.62.7.pss6207_0747>
9. Fleury MJ, Grenier G, Bamvita JM, Caron J. Professional service utilisation among patients with severe mental disorders. BMC health services research. 2010;10:141. Available from: <https://doi.org/10.1186/1472-6963-10-141>.
10. Giacco D, Bird VJ, Ahmad T, Bauer M, Lasalvia A, Lorant V, et al. The same or different psychiatrists for in- and out-patient treatment? A multi-country natural experiment. Epidemiology & Psychiatric Science. 2018;29:e10. Available from: [https://doi.org/[10.1017/S2045796018000732](https://doi.org/10.1017%2FS2045796018000732).](https://doi.org/10.1017/S2045796018000732)
11. Green CA, Polen MR, Janoff SL, Castleton DK, Wisdom JP, Vuckovic N, et al. Understanding how clinician-patient relationships and relational continuity of care affect recovery from serious mental illness: STARS study results. Psychiatric Rehabilitation Journal. 2008;32(1):9- 22. Available from: <https://doi.org/10.2975/32.1.2008.9.22>.
12. Hoertel N, Limosin F, Leleu H. Poor longitudinal continuity of care is associated with an increased mortality rate among patients with mental disorders: results from the French National Health Insurance Reimbursement Database. European Psychiatry: the Journal of the Association of European Psychiatrists. 2014;29(6):358-64. Available from: <https://doi.org/10.1016/j.eurpsy.2013.12.001>.
13. Kaltsidis G, Bamvita JM, Grenier G, Fleury MJ. Predictors of Frequent Emergency Department Utilization for Mental Health Reasons. Journal of Behavioral Health Services & Research. 2020;17:17. Available from: <https://doi.org/10.1007/s11414-020-09695-4>.
14. Macdonald A, Adamis D, Craig T, Murray R. Continuity of care and clinical outcomes in the community for people with severe mental illness. British Journal of Psychiatry. 2019;214(5):273-8. Available from: <https://doi.org/10.1192/bjp.2018.261>.
15. Mitton CR, Adair CE, McDougall GM, Marcoux G. Continuity of care and health care costs among persons with severe mental illness. Psychiatric Services. 2005;56(9):1070-6. Available from: <https://doi.org/10.1176/appi.ps.56.9.1070>.
16. Puntis SR, Rugkasa J, Burns T. The association between continuity of care and readmission to hospital in patients with severe psychosis. Social Psychiatry & Psychiatric Epidemiology. 2016;51(12):1633-43. Available from: <https://doi.org/10.1007/s00127-016-1287-3>.
17. Ride J, Kasteridis P, Gutacker N, Doran T, Rice N, Gravelle H, et al. Impact of family practice continuity of care on unplanned hospital use for people with serious mental illness. Health Services Research. 2019;54(6):1316-25. Available from: <https://doi.org/10.1111/1475-6773.13211>.
18. van der Lee A, de Haan L, Beekman A. Schizophrenia in the Netherlands: Continuity of Care with Better Quality of Care for Less Medical Costs. PLoS ONE [Electronic Resource]. 2016;11(6):e0157150. Available from: <https://doi.org/10.1371/journal.pone.0157150>.
19. Watkins KE, Paddock SM, Hudson TJ, Ounpraseuth S, Schrader AM, Hepner KA, et al. Association Between Quality Measures and Mortality in Individuals With Co-Occurring Mental Health and Substance Use Disorders. Journal of Substance Abuse Treatment. 2016;69:1-8. Available from: <https://doi.org/10.1016/j.jsat.2016.06.001>.
