# Supplementary material for: Relational continuity may give better clinical outcomes in patients with serious mental illness – a systematic review
Source: BMC Psychiatry. 2023 Dec 18;23:952. doi: 10.1186/s12888-023-05440-1 (PMC10729558; doi:10.1186/s12888-023-05440-1)
Supplement: Supplementary file 4 — Supplementary Material 4: Excluded studies [file 12888_2023_5440_MOESM4_ESM.docx]

# **Additional file 4** **- Excluded studies**

| **References** | **Main reason for exclusion** |
| --- | --- |
| Aalto A-M, Elovainio M, Vehko T, Sinervo T. Experiences of health services among patients with chronic conditions. *Int J Integr Care*. 2018;18:1-2. | Wrong publication type |
| Barekatain M, Maracy MR, Rajabi F, Baratian H. Aftercare services for patients with severe mental disorder: A randomized controlled trial. *J Res Med Sci.* 2014;19:240-245. | Wrong exposure |
| Bauer MS, McBride L, Williford WO, et al. Collaborative care for bipolar disorder: part I. Intervention and implementation in a randomized effectiveness trial. *Psychiatr Serv.* 2006;57:927-936. | Wrong exposure |
| Bauer MS, McBride L, Williford WO, et al. Collaborative care for bipolar disorder: Part II. Impact on clinical outcome, function, and costs. *Psychiatr Serv*. 2006;57:937-945. | Wrong exposure |
| Chaiyachati KH, Gordon K, Long T, et al. Continuity in a VA patient-centered medical home reduces emergency department visits. *PLoS One.* 2014;9:e96356. | Wrong population |
| Cheng SH, Chen CC, Hou YF. A longitudinal examination of continuity of care and avoidable hospitalization: evidence from a universal coverage health care system. Arch Intern Med. 2010;170:1671-1677. | Wrong population |
| Chopra I, Wilkins TL, Sambamoorthi U. Ambulatory Care Sensitive Hospitalizations among Medicaid Beneficiaries with Chronic Conditions*. Hosp Pract (1995).* 2016;44:48-59. | Wrong population |
| Coleman EA, Eilertsen TB, Kramer AM, Magid DJ, Beck A, Conner D. Reducing emergency visits in older adults with chronic illness. A randomized, controlled trial of group visits. *Eff Clin Pract.* 2001;4:49-57. | Wrong population |
| Colligan EM, Pines JM, Colantuoni E, Wolff JL. Factors Associated with Frequent Emergency Department Use in the Medicare Population. *Med Care Res Rev.* 2017;74:311-27. | Wrong population |
| Dreiher J, Comaneshter DS, Rosenbluth Y, Battat E, Bitterman H, Cohen AD. The association between continuity of care in the community and health outcomes: A population-based study. *Isr J Health Policy Res*. 2012;1:21. | Wrong population |
| Druss BG, Rohrbaugh RM, Levinson CM, Rosenheck RA. Integrated medical care for patients with serious psychiatric illness: a randomized trial. *Arch Gen Psychiatry.* 2001;58:861-868. | Wrong population |
| Gentil L, Huynh C, Grenier G, Fleury MJ. Predictors of emergency department visits for suicidal ideation and suicide attempt. *Psychiatry Res.* 2020;285:112805. | Wrong exposure |
| Gill JM, Mainous AG, Nsereko M. The effect of continuity of care on emergency department use*. Arch F Medicine*. 2000;9:333-338. | Wrong population |
| Greenberg GA, Rosenheck RA. Continuity of care and clinical outcomes in a national health system*. Psychiat Serv*. 2005;56:427-433. | Wrong exposure |
| Huisman A, Kerkhof AJ, Robben PB. Suicides in users of mental health care services: treatment characteristics and hindsight reflections. *Suicide Life Threat Behav*. 2011;41:41-49. | Wrong population |
| Hvid M, Vangborg K, Sørensen HJ, Nielsen IK, Stenborg JM, Wang AG. Preventing repetition of attempted suicide--II. The Amager project, a randomized controlled trial. *Nord J Psychiatry*. 2011;65:292-298. | Wrong population |
| Ionescu-Ittu R, McCusker J, Ciampi A, et al. Continuity of primary care and emergency department utilization among elderly people. *CMAJ*. 2007;177:1362-1368. | Wrong population |
| Kim W, Jang SY, Lee TH, Lee JE, Park EC. Association between continuity of care and subsequent hospitalization and mortality in patients with mood disorders: Results from the Korea National Health Insurance cohort  *PLoS One*. 2018;13:e0207740.  Macdonald A, Adamis D, Craig T, Murray R. Continuity of care and clinical outcomes in the community for people with severe mental illness. *Br J Psychiatry*. 2019;214:273-278. | Wrong population  Wrong population |
| McCusker J, Tousignant P, Borgès Da Silva R, et al. Factors predicting patient use of the emergency department: a retrospective cohort study. *CMAJ*. 2012;184:E307-316. | Wrong population |
| Novakovic V, Barkin RL, Hamner MB, LeClear O'Connell K. Schizophrenia: improving the continuity of care to ensure optimal patient outcomes. *Dis Mon*. 2012;58:395-409. | Wrong publication type |
| Rost K, Pyne JM, Dickinson LM, LoSasso AT. Cost-effectiveness of enhancing primary care depression management on an ongoing basis. *Ann Fam Medicine.* 2005;3:7-14. | Wrong population |
| Roux P, Passerieux C, Fleury MJ. Mediation analysis of severity of needs, service performance and outcomes for patients with mental disorders. *Br J Psychiatry*. 2016;209:511-516. | Wrong outcome |
| Schaefer JA, Cronkite RC, Hu KU. Differential relationships between continuity of care practices, engagement in continuing care, and abstinence among subgroups of patients with substance use and psychiatric disorders. *J Stud Alcohol Drugs*. 2011;72:611-621. | Wrong population |
| Sridhara S. Continuity of care for schizophrenia treatment in Maryland medicaid. *ProQuest. Information & Learning*. 2011. | Wrong publication type |
| Termorshuizen F, Van Der Hoorn BEA, Blanken P, Van Hemert AM, Hoek HW, Hendriks VM. Continuity of care and risk of hospital readmission among dual diagnosis patients. *Ment Health Subst Use*. 2012;5:20-30. | Wrong exposure |
| Theodoridou A, Hengartner MP, Gairing SK, Jager M, Ketteler D, Kawohl W, et al. Evaluation of a new person-centered integrated care model in psychiatry. *Psychiatr Q*. 2015;86:153-168. | Wrong exposure |
| Toulany A, Stukel TA, Kurdyak P, Fu L, Guttmann A. Association of Primary Care Continuity With Outcomes Following Transition to Adult Care for Adolescents With Severe Mental Illness*. JAMA Netw Open*. 2019;2:e198415. | Wrong population |
| van der Lee APM, de Haan L, Beekman ATF. Rising co-payments coincide with unwanted effects on continuity of healthcare for patients with schizophrenia in the Netherlands. *PLoS One*. 2019;14:e0222046. | Wrong exposure |
| Vita A, Corsini P, Bonomi S, Sacchetti E, Cesana BM. Factors affecting antipsychotic drug discontinuation in the treatment of schizophrenia: Evidence from a naturalistic, retrospective, 18-month follow-up study. *Schizophr Res*. 2008;104:302-304. | Wrong publication type |
| Wang C, Kuo HC, Cheng SF, Hung JL, Xiong JH, Tang PL. Continuity of care and multiple chronic conditions impact frequent use of outpatient services. *Health Informatics J*. 2020;26:318-327. | Wrong population |
| White ES, Pereira Gray D, Langley P, Evans PH. Fifty years of longitudinal continuity in general practice: a retrospective observational study*. Fam Pract.* 2016;33:148-153. | Wrong exposure |
| Wolinsky FD, Bentler SE, Liu L, et al. Continuity of care with a primary care physician and mortality in older adults. *J Gerontol A Biol Sci Med Sci*. 2010;65:421-428. | Wrong population |
| Ye M, Guo J, Song C, Zheng F. Effects of Out-of-hospital Continuing Nursing on Schizophrenia Patients' Rehabilitation and Quality of Life. *Open Med Wars*. 2017;12:501-505. | Wrong exposure |
| Yoon J, Cordasco KM, Chow A, Rubenstein LV. The relationship between same-day access and continuity in primary care and emergency department visits. *PLoS ONE*. 2015;10(9):e0135274. | Wrong population |
| Zeber JE, Copeland LA, Good CB, Fine MJ, Bauer MS, Kilbourne AM. Therapeutic alliance perceptions and medication adherence in patients with bipolar disorder. *J Affect Disorders*. 2008;107:53-62. | Wrong exposure |
