# Supplementary material for: Relational continuity may give better clinical outcomes in patients with serious mental illness – a systematic review
Source: BMC Psychiatry. 2023 Dec 18;23:952. doi: 10.1186/s12888-023-05440-1 (PMC10729558; doi:10.1186/s12888-023-05440-1)
Supplement: Supplementary file 5 — Supplementary Material 5: Detailed table of the included studies [file 12888_2023_5440_MOESM5_ESM.docx]

**Additional file 5 - Detailed table of the included studies**

| **Author**  **Year**  **Ref #** | Adair et al.  2005  [1] |
| --- | --- |
| **Study design**  **Country**  **Population**  **Setting** | Cohort study, 17-month follow-up 2001 to 2002.  Canada, Alberta.  486 patients with severe mental illness (65% mood disorders, 35% psychotic disorder) from three health regions were followed over a 17-month period). Mean age 42.5 (SD 10) years; 60% women.  Both in- and outpatient clinics. |
| **Exposure/ intervention** | Patient and observer rated continuity of care, using the Alberta Continuity of Services Scale for Mental Health (ACSS-MH). |
| **Outcome**  **Type of analysis**  **Confounders/ covariates in analysis** | EQ-5D (both the five-item index score and the 100-point visual analogue scale score) for generic quality of life.  Multiple linear regression.  Bivariate analysis, analysis of variance (of relevance for health economic assessment)  Final model 1 adjusted for income and problem severity at baseline and final model 2 adjusted for primary diagnosis, age, suicidality, income. |
| **Results** | Associations between ACSS-MH scores and EQ-5D:  **Model 1: EQ-5D visual analogue scale**  Patient related continuity score: b= 0.22 (95% CI 0.123 to 0.317), beta*=0.225, p<0.001  **Model 2: EQ-5D index score**  Observer related continuity score: b=0.008 (95% CI 0.005 to 0.012), beta*=0.263 p<0.001  Bivariate associations between quartiles of *patient* *rated* ACSS-MH and EQ-5D Index score:  Quartile mean (SD): 1 0.48 (0. 33) 2 0.57 (0.30) 3 0.60 (0.29) 4 0.62 (0.31) P for group comparison <0.01  Bivariate associations between quartiles of *observer rated* ACSS-MH and EQ-5D Index score: 1 0.41 (0.34) 2 0.62 (0.26) 3 0.62 (0.33) 4 0.62 (0.28) P for group comparison <0.001  Bivariate associations between quartiles of *patient* *rated* ACSS-MH and EQ 5D 100 VAS score: 1 56.0 (18) 2 60.2 (21) 3 66.4 (16) 4 68.3 (19) P for group comparison <0.001  Bivariate associations between quartiles of *observer rated* ACSS-MH and EQ 5D 100 VAS score: 1 55.1 (21) 2 62.5 (19) 3 66.4 (17) 4 66.6 (17) P for group comparison <0.001 |
| **Risk of bias**  **Comments** | High.  Almost same study sample as Mitton et al. 2005.  Authors tested two models for each continuity scale because they were only moderately correlated with each other (r=0.36, p<0.001). The reasons for testing different scales for the different outcomes were not motivated. |
|  |  |
| **Author**  **Year**  **Ref #** | Adnanes et al.  2019  [2] |
| **Study design**  **Country**  **Population**  **Setting** | National cross-sectional survey, comparing SMI to non-SMI (SMI= severe mental illness). Data obtained 2013.  Norway.  Population: 835 mental health outpatients with severe and not severe mental illness. Persons with severe mental illness (n=155) were diagnosed with schizophrenia, schizoaffective disorder or bipolar affective disorder. Age groups of persons with SMI: 18-23 7.0%, 24-29 16.2%, 30-39 24.3%, 40-49 27.6%, 50-59 14.6%, ≥60 10.3%, 63.9% women). Population selection of questionnaire responders, total national mapping population: n=23 167.  Patients receiving specialist outpatient psychiatric treatment. |
| **Exposure/ intervention** | Perception of CoC using the CONTINU-UM measure. * |
| **Outcome**  **Type of analysis**  **Confounders/ covariates in analysis** | QoL with the Manchester Short Assessment of Quality of Life (MANSA) questionnaire.  Linear multivariate regression between CoC and QoL.  Gender, age, education, income, living situation, contact with family, contact with friends, therapeutic relationship, unmet need for treatment, unmet need for activity. |
| **Results** | SMI patients’ CoC were positively associated with QoL  Linear regression coefficient: b=0.268 (95 % CI 0.070 to 0.466), p=0.008 |
| **Risk of bias**  **Comments** | Moderate  Note: cross sectional design, population selection of earlier questionnaire responders.  Authors investigated associations and do not claim causality in findings.  Exposure does not directly measure relational continuity to one professional (see expiation of exposure below) rather team continuity.  Self-reported outcome. |
| *Comment: The CONTINU-UM measure is based on 17 different domains also comprising assessments of importance, ease to access and satisfaction with domains. Only 13 of the 17 domains were included in analysis because most responders deemed the excluded domains irrelevant in their response. Total score of CoC measure used in the analysed ranged from 1-5. | |
|  |  |
| **Author**  **Year**  **Ref #** | Bindman et al.  2000  [3] |
| **Study design**  **Country**  **Population**  **Setting** | Prospective cohort study over 20 months after baseline interview, unclear date for exact study duration, likely end of 90´s.  England, South London area.  100 patients (mean age 41 years, 42% women) with severe mental illness (schizophrenia, schizoaffective disorder, bipolar affective disorder, or recurrent depressive disorder) having had two or more lifetime admissions to hospital.  All patients had contact with a general adult sector psychiatric teams in South London |
| **Exposure/ intervention** | Continuity of contact with particular professionals, operationalized as the number of community `keyworkers´ (an individual member of the mental health team identified as having principal responsibility for ensuring delivery of care) over a period of time. |
| **Outcome**  **Type of analysis**  **Confounders/ covariates in analysis** | BPRS (Brief psychiatric Rating Scale).  HoNOS (Health of the Nation Outcome Score).  GAF (Global Assessment of Functioning).  Multivariate linear regression  Age, sex, whether white, whether currently living alone or living in supported accommodation, time since onset of illness, whether diagnosed as schizophrenia; and symptom and function GAF scores and total HoNOS scores at baseline. Stepwise selection was used to determine variables remaining in final model. |
| **Results** | Number of keyworkers / months on:  Total HoNOS score: b coefficient - 0.07 (95% CI -0.14 to - 0.002), p=0.04  GAF disability score b coefficient -0.02 (95 % CI -0.04 to 0.002), p=0.09  BPRS did not remain in model after stepwise selection and results were not reported. |
| **Risk of bias**  **Comments** | Moderate  Small sample, attrition. Study´s main focus was changes in continuity and individual outcomes over time, rather than investigating effects of continuity of care. |
|  |  |
| **Author**  **Year**  **Ref #** | Catty et al.  2013  [4] |
| **Study design**  **Country**  **Population   Setting** | Prospective cohort study over 2 years, full study took place during 2002-2007.  England.  180 persons 18-65 years of age (mean age 43.1 year, SD 10.9, 44.4% women) with a long-term psychotic disorder and been in contact with psychiatric services for minimum 2 years.  Psychiatric services within seven community health teams in two mental health trusts the Care Programme Approach indicating allocation to a key worker or case manager. |
| **Exposure/ intervention** | CONTINU-UM was used to measure user rated overall “experienced” continuity.  Of the 7 care factors in CONTINU-UM Experience & Relationship, Regularity and Consolidation are of relevance to relational continuity. |
| **Outcome**  **Type of analysis**  **Confounders/ covariates in analysis** | BPRS: Brief Psychiatric Rating Scale.  Overall functioning: GAF (Global Assessment of Functioning)  Quality of life: MANSA and SEIQoL  Linear regression analysis.  Tested variables: time-point, mental health trust, team, gender, total number of lifetime admissions, type of accommodation, living situation, ethnic group, education, employment, informal carer, use of depot medication, alcohol or drugs, whether hospitalized in the previous year, age, duration of illness, functioning, symptomatology, empowerment and quality of life. |
| **Results** | Having a higher Experience & Relationship, score was associated with an increase in symptomology during the subsequent year (beta coefficient= 0.69 (95% CI 0.28 to 1.1)  Users with higher Regularity scores was more likely to be hospitalized in the subsequent year, OR = 1.166 (95% CI 0.977 to 1.393)  There were no other significant associations. |
| **Risk of bias**  **Comments** | High  High attrition. Note, unclear which variables were adjusted for in final model. Authors do not seem to consider them confounders. |
|  |  |
| **Author**  **Year**  **Ref #** | Chien et al.  2000  [5] |
| **Study design**  **Country**  **Population**  **Setting** | Register study using interview and claims data from Medicaid recipients.  USA, Maryland.  351 in- and outpatient with schizophrenia in the State of Maryland. Interviews performed in 1995 and sample included individuals with mental illness diagnosis or mental health service utilization between 1992-1993.  Age 18-64, presented in age groups, 51.6% women.  Medicaid recipient, in- and outpatient care. |
| **Exposure/ intervention** | Continuity of care (COC)  Usual provider continuity (UPC)  Sequential continuity (SECON) |
| **Outcome**  **Type of analysis**  **Confounders/ covariates in analysis** | Medicaid payments for mental illness care and total payments.  General life satisfaction, and satisfaction with health (2 of 10 Lehman Quality of Health domains).  The respondent rated quality of life dimensions on a scale from 1 (terrible) to 7 (delighted). General life satisfaction.  Satisfaction with health was based on six questions whose responses were averaged to obtain an overalls score  Linear regression model  Gender, age, race, education, monthly income living arrangements, location (Baltimore or Eastern Shore), marital status, contact with family, social contact, SPMI category 1, disability entitlement, mental illness hospitalization in previous year, Colorado Symptom Scale for depressive symptoms and for psychotic symptoms, presence of medical comorbidities, presence of mental comorbidities, screen for substance abuse problems, organizational type of usual source for medical care, organizational type of care source for mental health, MAC provider used for medical, and MAC provider used for mental health. In the Medicaid cost regressions, Medicaid costs from prior year included as covariate. |
| **Results** | **Regression results on general life satisfaction:**  UPC: b 0.171, p=n.s.  SECON: b 0.367, p=n.s.  **Regression results on satisfaction with health:**  UPC: b 0.208, p=n.s.  SECON: b 0.236, p=n.s.  Provider continuity was not not found to be related to general life satisfaction with health.  **Regression results on costs:**  Higher provider continuity was found to be related to lower costs and to lower likelihood of mental illness hospitalization. Provider continuity was not significantly related to general life satisfaction or to satisfaction with health.  Regression results for total Medicare costs:  UPC: b 8 909.83 USD/year (p<0.05)  SECON: b 12 959 USD /year (p<0.05)  Total Medicaid payments (mean USD 11,444) and mental health payments (mean USD 6,142) were significantly lower for persons with greater continuity experience during the year, for both UPC and SECON. A 10% increase in UPC was associated with USD 891 lower total Medicaid annual payments per person-year and USD 725 lower mental health payments. For SECON, a 10% increase in follow-up visits to the same provider was associated with a decrease in total Medicaid payments of USD 1,296 and in mental health payments of USD 924.  (Due to the almost-perfect correlation of COC and UPC, the subsequent analysis used the UPC and SECON measures only) |
| **Risk of bias**  **Comments** | Moderate  Note: very extensive adjustment, possible over adjustment. |
| USD = US dollar | |
|  |  |
| **Author**  **Year**  **Ref #** | Conti et al.  2012  [6] |
| **Study design**  **Country**  **Population**  **Setting** | Register study, with a 12-month follow-up period using data from regional psychiatric information system. Data obtained 2007.  Italy, Lombardy region.  A total of 11 797 patients, followed in the specialist mental healthcare system, who started a new pharmacological treatment for depression (n=5 851, mean age 49,8 years SD, women 66.1%), schizophrenia (n=4 975, mean age 46.4, women 47.8%) or bipolar disorder (n=971, mean age 48.5, women 56.3%) during 2007.  Specialist mental health care |
| **Exposure/ intervention** | Continuity of care was defined as receiving at least one psychiatric contact every 90 days. |
| **Outcome**  **Type of analysis**  **Confounders/ covariates in analysis** | Prescription records of antidepressants, antipsychotics, mood stabilizers operationalized as time to lack of persistence with initial pharmacological treatment. It was defined as a gap of at least 30 days between subsequent medication fills.  Cox regression.  Age groups, gender, education, employment status, marital status, urbanicity, comorbidity, psychiatric hospitalization in the previous 5 years; substance-use disorder; continuity and intensity of psychiatric care received after treatment initiation |
| **Results** | Continuity of care on lack of persistence:  Depression: HR 0.89 (95 % CI 0.71–1.13)  Schizophrenia HR 0.70 (95 % CI 0.63–0.77)  Bipolar disorder: HR 0.84 (95% CI 0.64–1.09) |
| **Risk of bias**  **Comments** | Moderate  Note: very extensive adjustment, possible over adjustment.  Exposure not clearly relevant to relational continuity. |
|  |  |
| **Author**  **Year**  **Ref #** | Desai et al.  2005  [7] |
| **Study design**  **Country**  **Population**  **Setting** | Register study using data from VA health care system over the 4-year period from January 1, 1994, to December 31, 1998.  USA  The sample included all patients (n=121 933, mean age 48.2 years SD 11.7, women 5.6%) discharged with a diagnosis of major affective disorder, bipolar affective disorder, posttraumatic stress disorder (PTSD), or schizophrenia from psychiatric inpatient units in the VA health care system. Of 121 933 unique patients included in the sample, 3 588(2.9%) died within 1 year of discharge. Of those, 481 (0.4% of the total sample, 13.4% of deaths) died of suicide.  Psychiatric inpatients discharged from any of 128 U.S. Department of Veterans Affairs hospitals between 1994 to 1998. |
| **Exposure/ intervention** | Six variables reflected delivery of mental health care, one being: a measure of continuity of outpatient care after discharge—the number of 2-month periods in the 6 months after discharge in which the patient had at least two outpatient visits for his or her primary discharge diagnosis (range=0–3). |
| **Outcome**  **Type of analysis**  **Confounders/ covariates in analysis** | Suicide.  Multivariate logistic regression.  Age, gender, race, disability, distance to the VA, year of discharge, diagnosis, and discharge to the community. |
| **Results** | Continuity of care (reference: 3):  0 Rate ratio 1.06; p 0.84  1 Rate ratio 1.59; p <0.03  2 Rate ratio 1.01, p= 0.97  * |
| **Risk of bias**  **Comments** | Moderate  Exposure does not directly measure relational continuity. |
| *Authors state that results show that poor continuity of care was associated with higher suicide risk. However, those with no follow-up visits were at similar risk to those who had more than two visits. | |
|  |  |
| **Author**  **Year**  **Ref #** | Farley et al.  2011  [8] |
| **Study design**  **Country**  **Population**  **Setting** | Register study using data from North Carolina Medicaid and for the period 2001–2003.  USA, North Carolina  A total of 7 868 patients with schizophrenia were identified from North Carolina Medicaid records for the period 2001–2003.  Mean age for those with 1 prescriber 43.3 years, SD 10.5 (50% women), 2 prescribers 43.2 years, SD 10.7 (52% women), 3 prescribers 42.0 years, SD 10.9 (54% women), 4 prescribers or more 40.2 years, SD 11.3 (59% women).  Unclear. All patients were enrolled to Medicaid and were on antipsychotic drugs |
| **Exposure/ intervention** | Number of unique prescribers who provided schizophrenia medication. |
| **Outcome**  **Type of analysis**  **Confounders/ covariates in analysis** | Adherence measured by the medication possession ratio (MPR*) from Medicaid claims data, categorized into non-adherent, partially adherent, fully adherent and excess filler.  Multivariate logistic regressions.  Age, gender, race, comorbidity and in some analyses also switching drugs. |
| **Results** | Ordered logistic regression on adherence, by number of prescribers (1= ref)  2 OR 1.32 (95% CI 1.21 to 1.45)  3 OR 1.69 (95% CI 1.46 to 1.95)  >=4 OR 2.59 (95% CI 2.06 to 3.27)  ** |
| **Risk of bias**  **Comments** | Moderate  Exposure does not directly measure relational continuity. |
| * MPR measures refill behavior and according to authors, thus, represents medication taking, and disease control.  **According to authors: patients with more prescribers were significantly more likely than patients with one prescriber to switch medications for and to be either fully adherent or excess fillers. | |
|  | |
| **Author**  **Year**  **Ref #** | Giacco et al.  2018  [9] |
| **Study design**  **Country**  **Population**  **Setting** | 1-year prospective natural experiment during 2014-2017 comparing 1-year clinical outcomes of personal continuity and specialisation in routine care in a large-scale study across five European countries.  Belgium, England, Germany, Italy and Poland.  Psychiatric in-patients (n=7 302, mean age 42.4 years, SD 14.3, women 47.7%) clinically diagnosed with a psychotic, mood or anxiety/dissociative/stress-related/somatoform disorder. 6 369 (87.2%) included in follow-up analysis.  Folllow-up of personal continuity by the same psychiatrist or under the care of different specialization of psychiatrists for in- and outpatient treatment. |
| **Exposure/ intervention** | Personal continuity, i.e., a patient is under the care of the same psychiatrist for in- and out-patient treatment; or specialisation, i.e., a patient is under the care of different psychiatrists for in- and out-patient treatment. |
| **Outcome**  **Type of analysis**  **Confounders/ covariates in analysis** | Readmission to hospital within 1 year following the index admission, obtained from medical records in England and Italy and via phone or personal interviews in the other countries.  Mixed effect logistic regression model with a random effect for hospital.  Age, gender, diagnostic group, whether a patient has been previously admitted, severity of illness at baseline, social situation, formal status of the patient at baseline, length of stay in hospital and country. |
| **Results** | **Readmission to hospital:**  Personal continuity vs specialization: OR 1.08 (95% CI 0.94–1.25), p=0.28  Women: personal continuity vs specialization: OR 1.12 (95% CI 0.91–1.38), p=0.28  Men: personal continuity vs specialization: 1.03 (95% CI 0.84–1.27), p=0.78  Psychotic disorders: personal continuity vs specialization OR 1.07 (95% CI 0.86–1.32), p=0.55 |
| **Risk of bias**  **Comments** | Moderate  Whether personal continuity or specialisation was deployed may depend on unknown and/or unmeasured factors. Comparison is based on a natural experiment. |
|  |  |
| **Author**  **Year**  **Ref #** | Hoertel et al.  2014  [11] |
| **Study design**  **Country**  **Population**  **Setting** | Observational study, using data from French National Health Insurance reimbursement database. Patients were followed from 2007-2010.  France  Sample of 14 515 (33.0% 19-40 years, 56.3% 41-65 years, 10.7% 66 years and older, women 65.5%) from National Health Insurance database of persons with any mental disorder. Of these, a diagnosis was reported for 2 863 patients (19.8%) and of these 554 (3.8%) with schizophrenia; 832 (5.7%) with major depressive disorder and 303 (2.1%) with bipolar disorder.  General French metropolitan population, specialist care. |
| **Exposure/ intervention** | COC Index |
| **Outcome**  **Type of analysis**  **Confounders/ covariates in analysis** | All causes mortality  Cox proportional regression models  Age, gender, comorbidities and social status in first step, interaction variables by testing variable pairs in first model. |
| **Results** | Overall results (for total population) showed significant associations between COC and death:  HR 0.83 (95% CI 0.83 to 0.83), p<0.0001  Results for subgroups by psychiatric condition in sensitivity analyses:  Schizophrenia HR 0.87 (95% CI 0.83–0.92), p<0.0001  Major depressive disorder HR 0.87 (95% CI 0.83–0.91), p<0.0001  Bipolar disorder HR 0.84 (95% CI 0.79–0.89), p<0.0001 |
| **Risk of bias**  **Comments** | Moderate |
|  |  |
| **Author**  **Year**  **Ref #** | Kaltsidis et al.  2020  [12] |
| **Study design**  **Country**  **Population**  **Setting** | Retrospective observational study of medical records and interview data over the 12 months prior to interview at the emergency department. Medical records obtained for 2016 to 2017.  Canada, Quebec  Population of n=320 (mean age 38.9 years, SD 13.6, women 51.6%) visiting emergency department for mental health reasons.  In- and outpatient specialist care. |
| **Exposure/ intervention** | Study investigates predictors of frequent emergency department utilization for mental health reasons. Factors were organized as predisposing, enabling and needs factors.  Within enabling factors: a regular source of care (outside the ED or hospitalization) over the 12 months prior to interview was regarded as relevant to CoC and was measured through health records. |
| **Outcome**  **Type of analysis**  **Confounders/ covariates in analysis** | Number of emergency department visits for mental health reasons over the 12 months prior to interview at the ED  Bivariate analyses were used to assess associations (with the alpha value set at p<0.10) between each independent variable and the dependent variable, separately. Multivariate hierarchical linear regression. Three analyses were performed for significantly associated variables introduced by blocks using backward elimination.  Final model adjusted for: Needs factors (diagnoses), Predisposing factors (frequency of past hospitalizations for mental health reasons) and Enabling factors ((having regular care from family physician or outpatient psychiatrist). |
| **Results** | Adjusted results: Having regular care from an outpatient psychiatrist (outside ED or hospitalization) over the 12 months prior to interview at the ED: beta: 0.123, p=.002488  Having regular care from an outpatient psychiatrist over the 12 months prior to interview at the ED was the only predictor of frequent ED utilization. |
| **Risk of bias**  **Comments** | Moderate  Adjustment for many covariates, exposure of interest is predictor in analysis and cannot be interpreted causally. Exposure does not directly measure relational continuity. |
| *p-value is calculated by the authors fof the pape from a reported t-value of 3.049, using 319 as degrees of freedom and a two-tailed test. | |
|  |  |
| **Author**  **Year**  **Ref #** | Macdonald et al.  2019  [13] |
| **Study design**  **Country**  **Population**  **Setting** | Register study using data from 2006–2016 obtained from the electronic patient record system held by the mental health trust.  England, South London  Patients (n=5 552, mean age 46.5 years, SD 16.8, 37,5% women) with schizophrenia or delusional disorder.  Psychiatric community out-patient teams. |
| **Exposure/ intervention** | Modified Modified Continuity Index (MMCI, range 0-1) measuring the number of teams caring for the patient over time. |
| **Outcome**  **Type of analysis**  **Confounders/ covariates in analysis** | HoNOS, Health of the Nation Outcomes Scales  The generalized estimating equations (GEE) method for longitudinal data. The estimated coefficients reflect the relationship between the longitudinal development of the dependent variable and the longitudinal development of the predictor variables, using all data.  Gender, age, ethnicity, number of teams caring for the patient, main diagnosis and Index of Multiple Deprivation. |
| **Results** | MMCI predicts HoNOS in adjusted analysis:  B regression coefficient: −0.624, (95% CI −0.896 to −0.352), p<0.001. Cohen’s d = 1.75. |
| **Risk of bias**  **Comments** | High  Unclear adjustments. Authors hypothesized a decline over the follow-up period in CoC and HoNOS due to organizational changes. |
| **Author**  **Year**  **Ref #** | Mitton et al.  2005  [14] |
| **Study design**  **Country**  **Population**  **Setting** | Observational cohort study using administrative data for most cost items, supplemented by patient interviews.  Canada, Alberta.  486 patients with severe mental illness (65% mood disorders, 35% psychotic disorder) confirmed by using a structured diagnostic interview, the Mini International Neuropsychiatric Interview (MINI), were followed over a 17-month period (March 2001 to December 2002).  Age and sex not stated in present manuscript but in Adair et al. 2005 (same population), mean age was 42.5 years, SD 10, 60% women.  Both in- and outpatient care. |
| **Exposure/ intervention** | Patient and observer rated continuity of care, using the Alberta Continuity of Services Scale for Mental Health (ACSS-MH). |
| **Outcome**  **Type of analysis**  **Confounders/ covariates in analysis** | Costs from payer perspective, including inpatient care, emergency department visits, outpatient and community care, home service visits, laboratory tests, and medications.  One-way analysis of variance for differences in means across quartiles of observer-rated continuity of care.  Multiple linear regression for associations between continuity of care and different cost categories (only 2 shown).  Cost categories: total costs, hospitalisation costs, costs for community services, drug costs, and non-GP physician costs.  Age, household income, duration of illness, recruitment location, and suicidality. |
| **Results** | Differences in mean costs between lowest and highest quartiles of observer-rated continuity of care:  **Total costs**: $CAN 23 942 (SD 27 628) vs. $CAN 23 347 (SD 25 919), p=0.054  **Hospital costs**: $CAN 13 634 (SD 20 574) vs. $CAN 9 331 (SD 20 979), p=0.001  **Community costs**: $CAN 2 042 (SD 3313) vs. $CAN 5 056 (SD 7264), p=0.001  **Drug costs**: $CAN 3 166 (SD 5973) vs. $CAN 6 502 (SD 7250), p=0.001  **Non-GP physician costs**: $CAN 5 232 (SD 6019) vs. $CAN 2 457 (SD 3505), p=0.001  Adjusted linear regression:  Observer-rated continuity on log **hospital costs**: beta: –0.24 (95% CI –0.03 to –0.006)  Observer-rated continuity on log **community costs**: beta: 0.26 (95% CI 0.008 to 0.025 |
| **Risk of bias**  **Comments** | Moderate  Same study and same sample as Adair et al 2005. |
| GP = general practitioner; $CAN = Canadian dollar |  |
|  |  |
| **Author**  **Year**  **Ref #** | Puntis et al.  2016  [15] |
| **Study design**  **Country**  **Population**  **Setting** | 36-month prospective cohort study. Recruitment between 2008 to 2011. Follow up data from medical records.  England  323 patients (mean age 39.6 years, SD 11.4, women 32.5%) with a psychosis diagnosis, currently detained in hospital involuntarily.  Unclear |
| **Exposure/ intervention** | Average gap between face-to-face contacts  Number of 60-day gaps without contact  Number of different mental health professions seen  Number of care coordinators  Number of psychiatrists |
| **Outcome**  **Type of analysis**  **Confounders/ covariates in analysis** | Readmission to hospital  Time to readmission  Number of days in hospital.  Multivariate logistic regression for readmission outcome. Proportional hazard models for time to readmission outcome and negative-binomial model for number of days in hospital outcome.  Age, gender, ethnicity, and BPRS score. |
| **Results** | **Readmission**  **Average gap between face-to-face contacts: OR 0.956 (95% CI 0.922 to 0.990)**  **Number of 60-day gaps without contact: OR: 1.154 (95% CI 0.897–1.484)**  **Number of different mental health professions seen: OR 1.056 (95% CI 0.776 to 1.436)**  **Number of care coordinators OR 1.154 (95% CI 0.930 to 1.433)**  **Number of psychiatrists not analyzed /reported**  **Time to readmission:**  **Average gap between face-to-face contacts: HR 0.996 (95% CI 0.989 to 1.003)**  **Number of 60-day gaps without contact: HR 0.597 (95% CI 0.481 to 0.743)**  **Number of different mental health professions seen: HR 0.848 (95% CI 0.761 to 0.945)**  **Number of care coordinators: HR 0.541 (95% CI 0.435 to 0.673)**  **Number of psychiatrists: HR 0.923 (95% CI 0.777 to 1.097)**  **Number of days in hospital:**  **Average gap between face-to-face contacts IRR 0.966 (95% CI 0.956 to 0.976)**  **Number of 60-day gaps without contact: IRR 0.904 (95% CI 0.810 to 1.010)**  **Number of different mental health professions seen: IRR 0.861 (95% CI 0.743 to 0.997)**  **Number of care coordinators: IRR 1.157 (95% CI 1.053 to 1.271)**  **Number of psychiatrists not analyzed /reported** |
| **Risk of bias**  **Comments** | Moderate |
| OR = odd ration, HR = hazard ratio, IRR = incidence rate ratio, BPRS = Brief Psychiatric Rating Scale | |
|  |  |
| **Author**  **Year**  **Ref #** | Ride et al.  2019  [16] |
| **Study design**  **Country**  **Population**  **Setting** | Observational cohort study investigating associations between care in family practice and unplanned hospital visits, 2007-2014.  England  The sample consisted of 19 324 (50.2% women, age adults) individuals attending 215 practices, observed for 15.8 3‐month periods on average (range 1‐28 periods). Population had bipolar disorder (35.4%), Schizophrenia and other psychoses (53.1%) or both (11.5%).  Family physicians. |
| **Exposure/ intervention** | Three indices measuring different dimensions of family physician relational continuity:  The Continuity of Care (COC)  The Usual Provider of Care (UPC)  The Sequential Continuity (SECON)  Continuity indices were defined as low or high based on the median value of each index:  COC low (0‐0.35), high (>0.35)  UPC low (0‐0.67), high (>0.67)  SECON low (0‐0.17), high (>0.17). |
| **Outcome**  **Type of analysis**  **Confounders/ covariates in analysis** | Emergency department (ED) presentations, and unplanned admissions for SMI and ambulatory care‐sensitive conditions (ACSC). Outcomes investigated for moderate (3-5 visits) and high (6 visits o more) visit frequency.  Cox regression analyses, random effects models.  Age, gender, ethnicity, deprivation of the person's neighborhood of residence, history of smoking, number of Charlson Index comorbidities, comorbid depression, diagnostic subgroup and number of years since diagnosis. Treatment for SMI was included as a time‐varying variable indicating that the individual had been prescribed an antipsychotic drug at least once in the 12‐month lookback period prior to the current period. |
| **Results** | **ED presentations:**  COC (random effect model)  Moderate visit frequency (3‐5 visits), High COC index vs low COC index: HR 0.84, (95% CI 0.77‐0.91), p<0.001  High visit frequency (6 or more visits), High COC index vs low COC index, HR 0.86 (95% CI 0.80‐0.92), p<0.001  UPC  Moderate visit frequency (3‐5 visits), High UPC index vs low UPC index: HR 0.90 (95% CI 0.83‐0.98) p<0.05  High visit frequency (6 or more visits), High UPC index vs low UPC index, HR 0.97 (95% CI 0.89‐1.05), n.s  SECON  Moderate visit frequency (3‐5 visits), High SECON index vs low SECON index: HR 0.84, (95% CI 0.77 to 0.92) p<0.001  High visit frequency (6 or more visits), High SECON index vs low SECON index, HR 0.90 (95% CI 0.84 to 0.97), p<0.01  **SMI admission**  COC (random effect model)  Moderate visit frequency (3‐5 visits), High COC index vs low COC index: HR 0.98 (95% CI 0.82‐1.16), n.s.  High visit frequency (6 or more visits), High COC index vs low COC index, HR 0.94 (95% CI 0.82‐1.08), n.s  UPC  Moderate visit frequency (3‐5 visits), High UPC index vs low UPC index: HR 0.90 (95% CI 0.75 to 1.08), n.s  High visit frequency (6 or more visits), High UPC index vs low UPC index, HR 0.79 (95% CI 0.66 to 0.95), p<0.05  SECON  Moderate visit frequency (3‐5 visits), High SECON index vs low SECON index: HR 0.81 (95% CI 0.67 to 0.98) p <0.05  High visit frequency (6 or more visits), High SECON index vs low SECON index, HR 0.94 (95% CI 0.78 to 1.15), n.s.  **ACSC admission:**  COC (random effect model)  Moderate visit frequency (3‐5 visits), High COC index vs low COC index: HR 0.74, (95% CI 0.62‐0.88), p<0.001  High visit frequency (6 or more visits), High COC index vs low COC index, HR 0.71 (95% CI 0.61‐0.82), p<0.001  UPC  Moderate visit frequency (3‐5 visits), High UPC index vs low UPC index: HR 0.83 (95% CI 0.70 to 0.99) p<0.05  High visit frequency (6 or more visits), High UPC index vs low UPC index, HR 0.79 (95% CI 0.66 to 0.93) p<0.01  SECON  Moderate visit frequency (3‐5 visits), High SECON index vs low SECON index: HR 0.83 (95% CI 0.69 to 0.99) p<0.05  High visit frequency (6 or more visits), High SECON index vs low SECON index, HR 0.83 (95% CI 0.69 to 0.99) p<0.05 |
| **Risk of bias**  **Comments** | Moderate  Lots of tests, not adjusted for potential multiplicity. Many covariates used, possible over-adjustment. |
| ACSC ambulatory care‐sensitive conditions. n.s = not statistically significant using 5% threshold. | |
|  |  |
| **Author**  **Year**  **Ref #** | van der Lee et al.  2016  [17] |
| **Study design**  **Country**  **Population**  **Setting** | Retrospective register based cohort study using insurance data from patients over 2008–2011.  The Netherlands  7 392 patients under 70 years of age (mean age 43.3 years, women 39% at year 0) with schizophrenia in 2008, data from Computerized claims data of a Dutch Health Insurer.  Outpatient psychiatric treatment. |
| **Exposure/ intervention** | Continuity of elective psychiatric care:  The number of follow-up years of elective psychiatric care in 2009–2011 was calculated.  Continuous care group: patients with 3 years of elective psychiatric care  No treatment group: patients without elective psychiatric care  1-year treatment group: 1 year of elective psychiatric care  2-year treatment group: 2 years of elective psychiatric care |
| **Outcome**  **Type of analysis**    **Confounders/ covariates in analysis** | 1) Acute treatment events  2) Inpatient care and somatic care  3) Medical costs (psychiatric and somatic care)  For outcomes 1 and 2, descriptive proportions of care according to outcome categorizations over number of years with elective psychiatric care.  For outcome 3 average costs according to outcome categorizations over number of years with elective psychiatric care and effect size Cohen’s d for continuous care group with 3 years of elective psychiatric care versus other groups.  None identified. |
| **Results** | Outcome measurements:  Acute treatment 33% of the patients had acute treatment events or inpatient treatment in 2009–2014. The continuous care group with three years of treatment showed least of these outcomes, 25% of the patients had any of those treatments.  The groups with less years of treatment suffered more acute treatment events or inpatient treatment with 34% in the no treatment group, 52% in the one year group and 68% in the two year treAatment group.  The amount of somatic care demonstrated a strong positive relation with the number of years of elective psychiatric care.  The effect sizes for costs of psychiatric care between the continuous care group and the one and two years treatment group were medium to large and there was almost no effect for the no treatment group. The costs of somatic care showed a reverse pattern of effect sizes. The total costs showed medium effects between the continuous care group and the one year and two years treatment groups. |
| **Risk of bias**  **Comments** | High  Inadequate analysis strategy. Lots of tests, no adjustment confounders or multiplicity. |
|  |  |
| **Author**  **Year**  **Ref #** | Watkins et al.  2016  [18] |
| **Study design**  **Country**  **Population**  **Setting** | Retrospective cohort study of patients receiving care for mental illness or substance disorders within Veterans Administration between October 2006 and September 2007.  USA  Patients (n=144 045, mean age 52.2 years SD 10.6, women 5.5%) with co-occurring mental illness (schizophrenia, bipolar disorder type 1, post-traumatic stress disorder and major depression) and substance use disorders who received care for these disorders paid for by the Veterans Administration between October 2006 and September 2007.  Both in- and outpatient care. |
| **Exposure/ intervention** | Continuous care over time which was defined as receiving at least one diagnosis-related visit (either mental illness or substance use disorder) each quarter over a one-year period from any type of provider. |
| **Outcome**  **Type of analysis**  **Confounders/ covariates in analysis** | Mortality 12 and 24 months after the end of the observation period (main outcome).  Avoidable excess mortality number (the number of deaths that potentially could have been averted had the patient received the respective quality measure.)  Logistic regression models. Difference in mortality rates for avoidable excess mortality number.  Age, gender, racial/ethnic background, marital status, rural/urban location and whether the veteran had a service-connected disability for a mental or substance use disorder. |
| **Results** | **Quality measure continuity of care (quarterly visits) had about***  Mortality 12 months: OR of about 0.73 (95% CI 0.66 to 0.78) on mortality at 12 months  Mortality 24 months: OR of about 0.77 (95% CI 0.71 to 0.82) on mortality at 24 months  **Avoidable excess mortality:**  Mortality rate  12ths: 2.3% vs. 3.1% mortality rate in those with more vs less CoC resulting in and avoidable excess mortality number of 655.7.  24ths: 4.6% vs. 5.8% mortality rate in those with more vs less CoC resulting in and avoidable excess mortality number of 983.6. |
| **Risk of bias**  **Comments** | Moderate  Exposure does not directly measure relational continuity. |
| *visually assessed from forest plot graph | |
